# The dual nature of plant growth-promoting bacteria: Benefits, risks, and pathways to sustainable deployment

**DOI:** 10.1016/j.crmicr.2025.100421

**Published:** 2025-06-16

**Authors:** Hassan Etesami

**Affiliations:** Department of Soil Science, University of Tehran, Tehran, Iran

**Keywords:** Plant growth-promoting bacteria, Phytotoxic metabolites, Hormonal imbalances, Microbial diversity disruption, Horizontal gene transfer, Multi-omics technologies

## Abstract

Plant growth-promoting bacteria (PGPB) are pivotal in sustainable agriculture, enhancing crop productivity and reducing reliance on chemical inputs. However, their dual role as beneficial agents and potential stressors remains underexplored. This review examines the paradoxical adverse effects of PGPB, challenging the predominantly optimistic narrative surrounding their use. At the plant level, unintended consequences include hormonal imbalances (e.g., auxin-induced root inhibition), phytotoxic metabolite production (e.g., hydrogen cyanide), and trade-offs between growth and defense mechanisms. At the soil level, risks encompass disrupted microbial diversity, altered nutrient cycling, and horizontal gene transfer that may foster pathogenicity. These outcomes are driven by environmental factors (soil pH and moisture), host-specific interactions, and application practices. Mitigation strategies emphasize rigorous strain selection, optimized dosing, and integrated soil management to balance efficacy with ecological safety. Advances in multi-omics technologies and synthetic consortia design offer predictive insights into strain behavior, while long-term ecological assessments are critical to address legacy impacts. The review underscores the necessity of a nuanced, evidence-based approach to PGPB deployment, harmonizing agricultural benefits with environmental stewardship. By addressing knowledge gaps in microbial ecology and risk assessment, this work supports strategies prioritizing both agricultural resilience and soil biodiversity to ensure PGPB contribute sustainably to global food security.

## Highlights

• Context-dependent hormone overproduction (e.g., IAA) by PGPB can disrupt root architecture and trigger stress ethylene, depending on host sensitivity, bacterial strain, and concentration.
• Strain-specific phytotoxin production may damage non-target plants, exacerbated by environmental stressors like nutrient-poor soils.
• Inoculant-driven microbial community shifts can impair nutrient cycles, particularly in low-diversity soils or at high application densities.
• Horizontal gene transfer of virulence traits to PGPB in agricultural hotspots (e.g., rhizospheres) risks creating latent pathogens.
• Optimized strain selection via multi-omics (genomics/metabolomics) and field validation are critical to mitigate risks, given host-genotype specificity and environmental contingency.

## Graphical abstract

**Figure:**
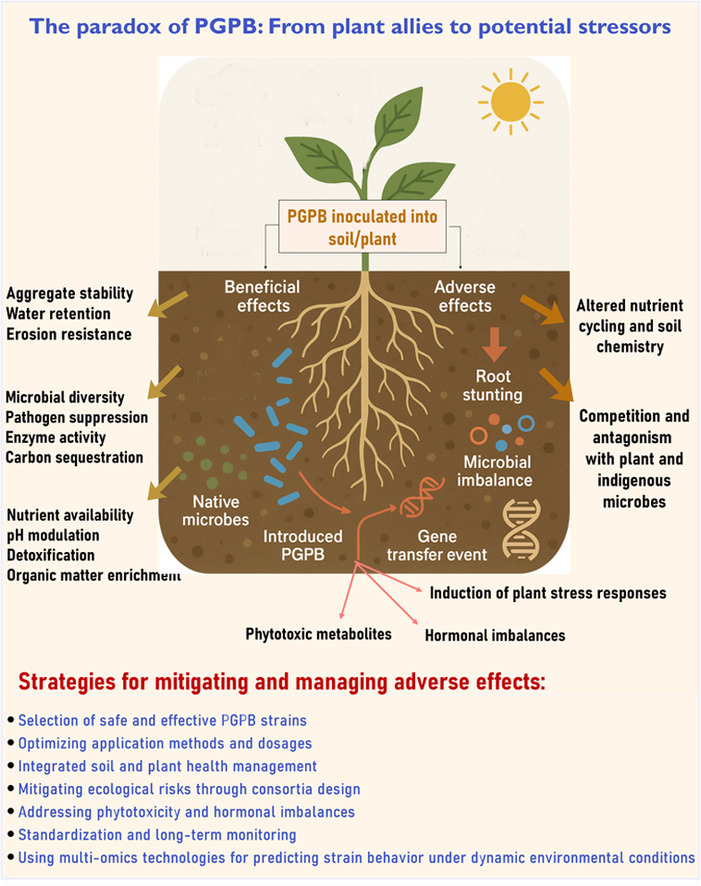

## 1 Introduction

Plant growth-promoting bacteria (PGPB) have emerged as a cornerstone of modern sustainable agriculture, offering innovative solutions to some of the most pressing challenges facing global food systems. These beneficial microbes play a pivotal role in enhancing crop productivity, reducing dependency on chemical fertilizers, and mitigating environmental degradation (Cao et al., 2023; Etesami and Maheshwari, 2018; Fiodor et al., 2021). Through diverse mechanisms such as nitrogen fixation, nutrient solubilization, phytohormone production, and biocontrol against pathogens, PGPB contribute to improved plant health and resilience (Ajijah et al., 2023; Ambrosini et al., 2016; Etesami et al., 2023; Etesami and Maheshwari, 2018). In recent years, the commercialization of PGPB-based biofertilizers and biostimulants has gained significant momentum, driven by the growing demand for eco-friendly agricultural practices. Governments, researchers, and industries worldwide are investing heavily in developing microbial technologies to address issues such as climate change, soil degradation, and food security (Al-Tammar and Khalifa, 2022; Joshi and Gauraha, 2022; Kumar et al., 2024). Despite these advancements, the narrative surrounding PGPB has largely been optimistic, focusing on their potential to revolutionize agriculture while overlooking potential risks or unintended consequences (e.g., antibiotic resistance genes, ecological disruption, regulatory and safety challenges, and field performance variability) (Bashan et al., 2014; Ferreira et al., 2019; Mahdi et al., 2022; Rilling et al., 2019).

While the benefits of PGPB are well-documented (Etesami and Maheshwari, 2018; Jeyanthi and Kanimozhi, 2018), there is a notable lack of attention to their potential adverse effects. This publication bias arises from several factors. First, research funding and industry priorities often favor studies that highlight success stories, leading to an overemphasis on positive outcomes in laboratory and greenhouse trials (Philippot et al., 2013). Second, negative results—such as failed field trials or unintended ecological impacts—are less likely to be published due to perceived lack of novelty or fear of undermining the credibility of PGPB technologies. As a result, the scientific literature presents a skewed picture of PGPB efficacy, creating a knowledge gap regarding their limitations and risks. Moreover, the complexity of plant-microbe interactions makes it challenging to isolate and interpret negative outcomes. Factors such as host genotype, soil type, environmental stressors, and microbial competition can significantly influence whether PGPB act as allies or adversaries (Ambrosini et al., 2016; Rilling et al., 2019). Without a comprehensive understanding of these dynamics, the full spectrum of PGPB impacts remains underexplored, leaving farmers, policymakers, and stakeholders ill-equipped to manage potential risks effectively. This review aims to address the critical need for a balanced perspective on PGPB by systematically examining their adverse effects on plants and ecosystems. While the benefits of PGPB are undeniable, understanding their limitations is equally important for ensuring their safe and responsible use in agriculture. This review provides a nuanced evaluation of PGPB performance in real-world conditions by synthesizing documented cases of harm, including phytotoxicity, disease promotion, and ecological disruption. The objectives of this review are threefold: i. To critically analyze the mechanisms through which PGPB may negatively impact plant health and soil ecosystems. ii. To identify the drivers of adverse outcomes, including strain misapplication, environmental modulators, and ecological interactions. iii. To propose strategies for mitigating risks and improving the reliability of PGPB technologies in sustainable agriculture. By bridging the gap between the perceived benefits and overlooked risks of PGPB, this review aims to foster a more holistic understanding of their role in agroecosystems. Ultimately, the goal is to empower researchers, practitioners, and policymakers to harness the potential of PGPB responsibly, ensuring that their contributions align with the broader goals of sustainability and food security.

## 2 Beneficial roles of PGPB

**Fig. 1:**
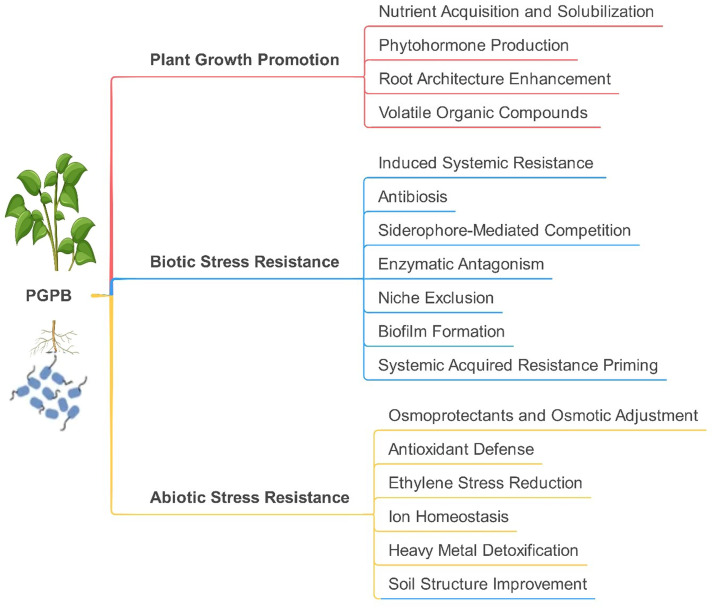
Mechanisms of plant growth-promoting bacteria (PGPB) in enhancing plant growth and stress resistance. PGPB enhance plant growth and resilience through multifaceted strategies. By solubilizing nutrients (e.g., nitrogen fixation, phosphate, and potassium mobilization) and producing phytohormones like auxins and cytokinins, PGPB directly stimulate root development and nutrient uptake. They mitigate abiotic stresses via osmoprotectants (proline and glycine betaine), antioxidant enzyme induction (SOD and CAT), and ACC deaminase activity to reduce ethylene-induced stress. Under biotic stress, PGPB suppress pathogens through antibiosis (antibiotics), siderophore-mediated iron competition, and induced systemic resistance (ISR) via jasmonic acid/ethylene signaling, while enzymes like chitinases degrade pathogen cell walls. Synergistically, improved root architecture, nutrient efficiency, and hormonal crosstalk amplify plant health, enabling adaptation to environmental challenges. These interconnected mechanisms underscore PGPB’s role in sustainable agriculture by reducing reliance on chemical inputs.

Plant Growth-Promoting Bacteria (PGPB), a term coined by Kloepper and Schroth (1981), encompass a diverse group of microorganisms that enhance plant growth through direct and indirect mechanisms, playing pivotal roles in sustainable agriculture (Fig. 1). These bacteria colonize the rhizosphere (rhizoplane), endosphere, and phyllosphere of plants, with densities up to a thousand times higher in the rhizosphere than bulk soil due to root exudates rich in carbon and metabolites (Dutta and Podile, 2010; Glick, 2014). PGPB are classified as extracellular, residing on root surfaces or intercellular spaces, or intracellular, forming symbiotic nodules, with modern criteria requiring aggressive colonization plus plant growth stimulation or biocontrol (Bhattacharyya and Jha, 2012; Martínez-Viveros et al., 2010). Direct mechanisms include nutrient acquisition via nitrogen fixation, potassium and phosphate solubilization, and siderophore-mediated iron uptake, alongside phytohormone production. Nitrogen-fixing genera like Rhizobium, Azotobacter, and Azospirillum convert atmospheric N₂ to ammonium, often forming symbiosomes in legume nodules to optimize nitrogenase activity (Etesami and Maheshwari, 2018; Olanrewaju et al., 2017). Phosphate-solubilizing bacteria (e.g., Bacillus and Pseudomonas) release insoluble soil phosphorus, while mycorrhizal helper bacteria like Streptomyces enhance fungal symbiosis for phosphorus uptake (Baba et al., 2015; Franco-Correa et al., 2010). Potassium-solubilizing bacteria can solubilize potassium-bearing minerals, converting insoluble potassium into soluble forms that are available for plant uptake (Etesami et al., 2017; Meena et al., 2016). Siderophores, low-molecular-weight Fe(III)-chelators produced by Azotobacter, Bacillus, and others, mitigate iron scarcity by solubilizing Fe from minerals, with concentrations peaking in the rhizosphere (10⁻⁴–10⁻³ M) compared to bulk soil (Ahmed and Holmström, 2014; Saha et al., 2016). These molecules, often hydroxamates or catecholates, form stable octahedral complexes, with photodegradation and mineral adsorption influencing Fe bioavailability (Barbeau et al., 2003; Hider and Kong, 2010). Phytohormones like indole-3-acetic acid (IAA), synthesized by Azospirillum and Bacillus via tryptophan-dependent pathways, regulate root architecture, though excessive IAA can inhibit growth, necessitating plant conjugation mechanisms (Etesami and Glick, 2024). Ethylene modulation via 1-aminocyclopropane 1-carboxylate (ACC) deaminase, produced by Pseudomonas and Azotobacter, cleaves the ethylene precursor ACC, alleviating stress-induced ethylene toxicity in drought, salinity, or heavy metals (Etesami and Glick, 2020; Glick, 2014). Indirectly, PGPB suppress pathogens through antibiotics (e.g., Bacillus), hydrogen cyanide (HCN) production, hydrolytic enzymes, and niche competition, enhancing biocontrol (Etesami et al., 2023). Collectively, these multifaceted interactions underscore PGPB’s integral role in enhancing plant resilience, nutrient efficiency, and yield, positioning them as vital agents in eco-friendly agricultural practices.

## 3 Constraints on PGPB in agriculture

**Fig. 2:**
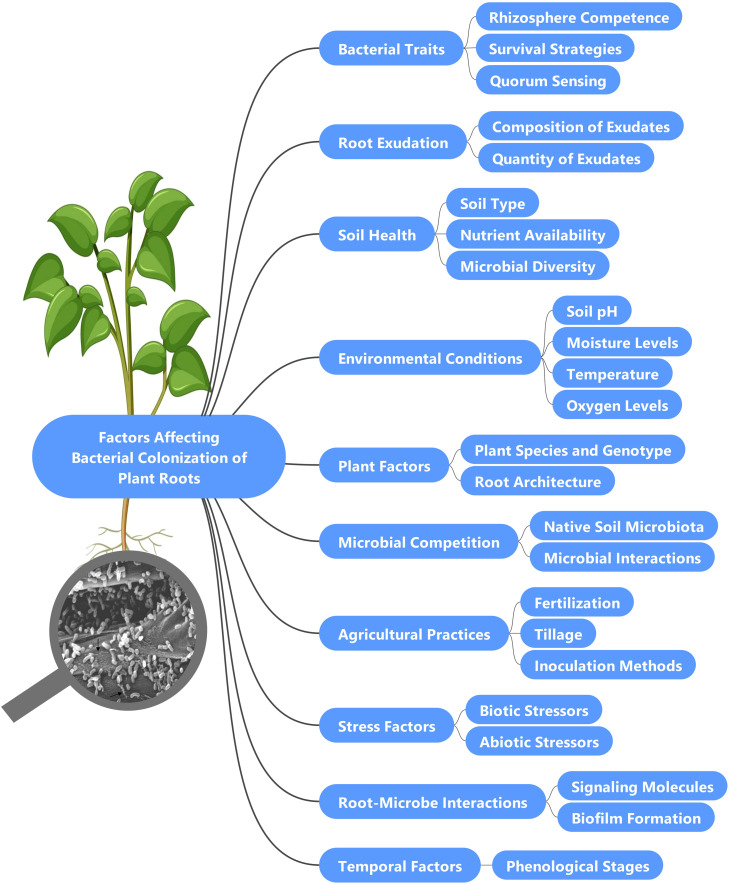
Factors affecting bacterial colonization of plant roots. Bacterial colonization of plant roots is influenced by a variety of interconnected factors. Key bacterial traits, such as rhizosphere competence and survival strategies, play a crucial role in their ability to establish on roots. The composition and quantity of root exudates, which include sugars, amino acids, and organic acids, significantly impact microbial attraction and growth. Soil health, characterized by soil type, nutrient availability, and microbial diversity, is essential for effective colonization. Environmental conditions, including soil pH, moisture levels, temperature, and oxygen availability, also affect bacterial activity. Additionally, plant species and genotype influence microbial community selection, while competition with native soil microbiota can limit the success of introduced plant growth-promoting bacteria (PGPB). Agricultural practices, such as fertilization and tillage, further alter microbial dynamics, and various stress factors, both biotic and abiotic, can hinder bacterial colonization. Root-microbe interactions, facilitated by signaling molecules and biofilm formation, enhance attachment to plant roots, while temporal factors related to the plant’s phenological stages can shape colonization patterns.

Agroecosystems are dynamic environments where the efficacy of PGPB is constrained by complex interactions among biotic and abiotic factors (Rilling et al., 2019) (Fig. 2). Plant traits, including genotype, developmental stage, and root exudate profiles, critically shape microbial colonization, as seen in the selective symbiosis between legumes and Rhizobium during nodulation (Depret and Laguerre, 2008). The rhizosphere, a hotspot for microbial activity, demands metabolic versatility from PGPB to compete with indigenous microbes, exemplified by *Pseudomonas putida* W619’s reduced colonization in unsterile versus sterile soils (Weyens et al., 2012). Synergistic interactions, such as those between *Pseudomonas striata* and the fungus *Piriformospora indica* in maize, highlight the role of microbial partnerships in enhancing colonization success (Singh et al., 2009). Soil chemistry and management practices further modulate PGPB outcomes: pH fluctuations can suppress bacterial growth (Fernández-Calviño and Bååth, 2010), while fertilization strategies—such as phosphorus and nitrogen inputs—may either boost colonization (e.g., *Pantoea agglomerans* and *Azotobacter chroococcum*; Narula et al. (2007) or hinder it (e.g., suppression of *Azospirillum* sp. under high nitrogen; Ramirez et al. (2012)). Tillage disrupts microbial networks (Bissett et al., 2013), and inoculation methods (e.g., carrier-based vs. seed coating) significantly impact survival rates, with lyophilized coatings improving maize seedling emergence by 92.5 % (Barra et al., 2016). Environmental stressors, including temperature extremes (Chen et al., 2021; Raza et al., 2022; Zahran, 1999), drought, and salinity (Ali et al., 2022; Serraj, 2003; Serraj et al., 1999), destabilize plant-microbe interactions, impairing nitrogen fixation and nodulation. Reactive oxygen species (ROS), though integral to signaling, become detrimental under stress (Abd-Alla et al., 2023; Lyu et al., 2022), while early nitrogen application alters root exudates, suppressing rhizobial colonization (Lyu et al., 2022). Traditional in vitro screening methods often fail to predict field performance, as seen with *Pantoea* sp. (I.059), which excels in lab settings but falters in soil due to niche competition (Chauhan et al., 2015; De Zutter et al., 2022). These challenges underscore the need for context-specific (outcomes varying with environmental/host variables) solutions, such as stress-adapted inoculants and multi-strain consortia, to enhance PGPB resilience in stressed agroecosystems (Maria Daniela Artigas and Jean Louise Cocson, 2023).

In summary, the efficacy of PGPB is constrained by interactions among biotic factors, such as host genotype and microbial competition, as well as abiotic factors, including soil chemistry, temperature, and moisture. The net benefit-risk balance relies on specific environmental thresholds. Benefit-dominant conditions include optimal soil moisture (60–80 % field capacity), moderate temperatures (20–30 °C), neutral pH (6.5–7.5), and low native microbial diversity, all of which enhance colonization and function (Duflos et al., 2024; Rilling et al., 2019; Yang et al., 2024). Conversely, risk-prone conditions involve drought or waterlogging, extreme temperatures (below 10 °C or above 40 °C), acidic or alkaline soils (pH below 5.5 or above 8.5), and high microbial competition, which can amplify unintended effects, such as auxin-induced phytotoxicity under drought conditions (Araujo et al., 2025; Duflos et al., 2024; Rilling et al., 2019). Therefore, the application of PGPB requires context-aware thresholds to maximize benefits while mitigating ecological trade-offs—resource reallocation compromising one function to enhance another. Addressing these constraints, which may inhibit the effectiveness of biofertilizers, necessitates the integration of microbial ecology with agricultural practices. This includes prioritizing rhizosphere competence and replicating field complexity in screening protocols to advance sustainable biofertilizer applications.

## 4 The paradox of PGPB: from plant allies to potential stressors

Emerging evidence reveals that PGPB have the potential to act as stressors under specific conditions emerging evidence reveals their potential to act as stressors under specific conditions (Table 1, Table 2). This paradox—the simultaneous occurrence of mutually exclusive outcomes—arises from their complex interactions with plants, soil microbiota, and environmental variables. Certain PGPB strains produce bioactive metabolites—such as antibiotics, volatile organic compounds (e.g., ammonia and dimethyl disulfide), hydrogen cyanide, or excessive phytohormones—that can shift from beneficial to detrimental depending on environmental context (Cordovez et al., 2018; Kai et al., 2010; Liu et al., 2024; Sehrawat et al., 2022; Wenke et al., 2012). For instance, overproduction of auxins or ethylene by PGPB may disrupt plant hormonal equilibrium, triggering stunted growth or tissue necrosis (Ali et al., 2009; Etesami and Glick, 2024). Similarly, the antibiotics synthesized to suppress pathogens (Etesami et al., 2023; Olanrewaju et al., 2017) might inadvertently damage plant cells when environmental stressors like drought or salinity compromise plant resilience (Khan et al., 2021). PGPB inoculation can also destabilize indigenous soil microbial communities, particularly in biodiversity-depleted ecosystems (Ambrosini et al., 2016; Gomes et al., 2005; Mawarda et al., 2020; Vuolo et al., 2022). For example, introduced strains may outcompete native arbuscular mycorrhizal fungi (AMF), which play pivotal roles in nutrient cycling and ecosystem stability, especially in semiarid Mediterranean regions (Requena et al., 1996; Van Der Heijden et al., 1998, 2006). Such disruptions risk cascading effects on plant diversity and soil health, particularly in degraded environments where microbial resilience is already compromised (Armada et al., 2018; Vuolo et al., 2022).

**Table 1:** Adverse effects of inoculated bacteria on plant growth.

Bacterial strain	Plant	Effect	References
Arthrobacter sp. (BS28–7) and Streptomyces alboflavus (BS43)	Maize (*Zea mays* L.)	Negative effects on total leaf area, shoot dry mass, and root dry mass; excessive biofilm production hindered plant development under drying-wetting cycles	Araújo et al. (2023)
*Bacillus mojavensis RRC101*	*Arabidopsis thaliana*	Phytotoxicity, including bleaching and reduced biomass, when grown on nutrient agar (NA) medium	Rath et al. (2018)
Pseudomonas aeruginosa PAO1	*Arabidopsis thaliana*	High cyanogenesis; significant plant growth inhibition and toxicity due to HCN production	Blom et al. (2011)
*Bacillus megaterium ORE8 and Pantoea* sp. *ORTB2*	Sweet pepper (Capsicum annuum)	Decreased radicle length, inhibiting growth	Samayoa et al. (2020)
Enterobacter sp. I-3	Radish	Significantly reduced shoot length, inhibiting growth	Park et al. (2015)
Enterobacter sp. I-3	Lettuce	Decreased leaf length, leaf width, and root length; increased lateral roots, inhibiting overall growth	Park et al. (2015)
Acinetobacter calcoaceticus P23 and Pseudomonas fulva Ps6	*Lemna gibba* (duckweed)	Inhibited growth, failed to promote growth; no significant inhibition observed	Khairina et al. (2021)
Serratia plymuthica and Pseudomonas brassicacearum	*Jacobaea vulgaris*	Reduced root growth (concentration-dependent); inhibited seed germination and seedling growth via VOCs	Liu et al. (2024)
*Bacillus amyloliquefaciens GB03*	*Arabidopsis thaliana*	Inhibition of plant growth at close distances (7 cm) due to pyrazine and 2,5-dimethylpyrazine	Song et al. (2021)

**Table 2:** Effect of inoculated bacteria on soil microbes/diversity.

Inoculated bacteria	Observed effects	References
*Pseudomonas aeruginosa* (strain GNS.13.2a)	Transient reduction in native bacterial CFU; native community rebounded within a week. Inoculant declined by >99 % due to native microbiota antagonism	Thomas and Sekhar (2016)
*Pseudomonas jessenii*	Altered bacterial community composition in lettuce rhizosphere, dependent on soil type and time	Schreiter et al. (2014)
*Pseudomonas aeruginosa*	Reduced invasiveness in wheat rhizosphere due to high native microbial diversity	Matos et al. (2005)
*Microbispora* sp.*, Streptomyces* sp., and *Nocardioides albus* (NutriLife 4/20)	Reduced actinobacterial diversity (40 to 21 genera) and colonization levels (14–86 %) in wheat roots	Conn and Franco (2004)
*Azospirillum lipoferum* CRT1	Increased variability in rhizobacterial genetic diversity in maize; no change in total abundance	Baudoin et al. (2009)
*Rhizobium leguminosarum* bv. *viciae*	Decreased bacterial diversity in faba bean rhizosphere, negatively correlated with microbial biomass	Zhang et al. (2010)
*Azospirillum brasilense* Sp6	Altered DGGE profiles of rhizosphere communities in mine tailings	De-Bashan et al. (2010)
*Sinorhizobium meliloti*	Shifted ammonia-oxidizing bacteria dominance (*Nitrosomonas* vs. *Nitrosospira*) in alfalfa	Sun et al. (2009)
*Sinorhizobium meliloti* (GM strain)	Horizontal plasmid transfer to non-target strains; persistence in soil for years	Morrissey et al. (2002)
*Azospirillum argentinense* Az39	Reduced bacterial community evenness in maize	Coniglio et al. (2022)
*Sinorhizobium meliloti* L33	Increased Alphaproteobacteria, reduced Gammaproteobacteria in alfalfa rhizosphere	Schwieger and Tebbe (2000)
*Bacillus amyloliquefaciens*	Gadhave et al. (2018)	Reduced *Pseudomonas* populations in broccoli endosphere
Consortium (*Ensifer, Acinetobacter,* and *Flavobacterium*)	Wang et al. (2018)	Reduced Actinobacteria and Firmicutes in cucumber rhizosphere
*Pseudomonas fluorescens* 2P24	Gao et al. (2012) and Trabelsi and Mhamdi (2013)	Transient alteration of fungal communities in cucumber rhizosphere

As mentioned above, PGPB performance is tightly linked to abiotic factors like soil pH, temperature, and moisture (Rilling et al., 2019). In degraded soils or under drought stress, plant-microbe interactions may falter, as stressed plants often prioritize resource allocation to defense over growth—a trade-off that diminishes PGPB benefits (Aboim et al., 2008; Peixoto et al., 2010; Ren et al., 2019). Furthermore, PGPB strains lacking adaptation to local conditions may fail to colonize roots effectively, underscoring the need for optimized strain selection (Compant et al., 2005). The paradox of PGPB highlights the delicate interplay between microbial benefits and ecological risks. Their successful application hinges on nuanced considerations: strain specificity, environmental compatibility, and plant physiological status. By prioritizing these factors, PGPB can be strategically harnessed as sustainable allies, minimizing unintended harm and maximizing agricultural resilience.

## 5 Adverse effects of PGPB on plant health

**Fig. 3:**
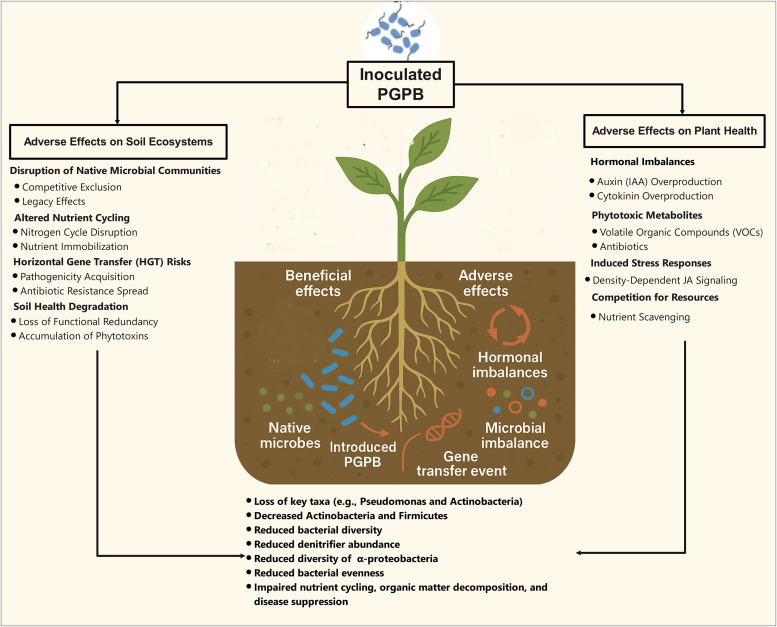
Adverse effects of plant growth-promoting bacteria (PGPB) on soil and plants. PGPB exhibit a paradoxical dual role in agroecosystems, with unintended adverse effects on plants and soil health. Hormonal imbalances arise from excessive auxin (IAA) or cytokinin production, disrupting root architecture and triggering stress ethylene synthesis, leading to stunted growth or leaf senescence. Phytotoxic metabolites, such as hydrogen cyanide (HCN) and volatile organic compounds (VOCs), inhibit mitochondrial function and induce oxidative stress, causing cell death. Resource competition between introduced PGPB and native microbes immobilizes nutrients (e.g., nitrogen and phosphorus), starving plants during critical growth stages. Soil microbial disruption occurs via competitive exclusion of beneficial taxa (e.g., mycorrhizal fungi and actinobacteria), reducing biodiversity and destabilizing nutrient cycling. Horizontal gene transfer (HGT) risks include the acquisition of virulence or antibiotic resistance genes, amplifying pathogenicity and resistance in soil communities. These effects are context-dependent, exacerbated by environmental stressors (e.g., drought and salinity), soil properties (e.g., low pH and high phosphorus), and host-specific mismatches. Mitigating these risks requires precision in strain selection, application timing, and integrated soil management to balance agricultural benefits with ecological resilience.

Numerous issues contribute to PGPB-based biofertilizers' low market share (Basu et al., 2021). Although a varied community of PGPB has been identified with versatile plant growth-promoting characteristics, very few PGPB are successfully registered for commercial use owing to inconsistent results in nursery/ greenhouse, and field-level experiments (Hossain et al., 2023). In some cases, the use of PGPB can have negative effects (Table 1). For example, individual inoculation of Arthrobacter sp. and its consortium with Streptomyces alboflavus showed a negative impact on the growth of maize under certain soil moisture conditions. The greatest total biomass was observed in uninoculated treatments compared to those inoculated with these bacteria under a water gradient treatment (Araújo et al., 2023). Samayoa et al. (2020) found that while certain strains like ORE8 (Bacillus megaterium) and ORTB2 (Pantoea sp.) promoted growth in onion, they inhibited the growth of sweet pepper seedlings. Another study mentioned that 7.5 % of observations reported negative effects of microbial inoculation on plant growth compared to uninoculated plants, although the majority (92 %) showed positive outcomes. This highlights that while inoculation generally benefits plant growth, there are exceptions where it may inhibit growth, possibly depending on factors like microbial strain, plant species, or environmental conditions (Dadzie et al., 2023). Beneficial bacteria can inadvertently weaken plant defense systems through diverse molecular and ecological mechanisms. According to the literature, PGPB primarily inhibit plant growth through mechanisms such as hormonal imbalances, the production of phytotoxic metabolites, and competition and antagonism with plant and indigenous microbes (Fig. 3).

### 5.1 Hormonal imbalances

Phytohormones are central to plant growth, development, productivity, and stress adaptation. Soil bacteria, particularly PGPB, influence phytohormone dynamics in plants and soil, directly modulating growth, development, and stress tolerance. These bacteria synthesize, degrade, or modulate phytohormones such as auxins, cytokinins, gibberellins, ethylene, and abscisic acid (ABA), either directly or via volatile organic compounds (VOCs) (Egamberdieva et al., 2017; Etesami and Glick, 2024; Orozco-Mosqueda et al., 2023; Santoro et al., 2011; Timofeeva et al., 2024). However, imbalances in phytohormone levels—whether from microbial production, degradation, or altered plant metabolism—can disrupt plant health (Dodd et al., 2010; Zhang et al., 2007). Since both plants and bacteria are capable of producing the hormones, the impact of their hormone production can lead to either positive or negative effects on plant growth. The negative effects arise when the combined hormone production from both bacteria and plants exceeds the optimal level required for plant growth. This overproduction can result in inhibitory effects, ultimately hindering plant development (Kudoyarova et al., 2019).

#### 5.1.1 Auxin (IAA)

The role of PGPB in enhancing crop growth through the production of phytohormones, particularly indole-3-acetic acid (IAA), has been extensively documented (Etesami and Glick, 2024). IAA plays a pivotal role in regulating various aspects of plant development, including root and shoot growth, cell expansion, vascular tissue differentiation, root bacterial colonization, pathogen defense, cell division stimulation, stem and root elongation, and loosening of root cell walls (Etesami and Glick, 2024; Orozco-Mosqueda et al., 2023). When bacterial IAA production synergizes with endogenous IAA synthesized by the plant, it can elevate overall IAA levels. However, excessive IAA concentrations may lead to growth abnormalities, such as stunted roots, reduced root hair formation, or even inhibition of shoot growth in extreme cases. For instance, Ali et al. (2009) demonstrated that bacterial IAA production could inhibit primary root elongation while promoting lateral root formation, highlighting the dual effects of IAA on root architecture. This underscores the importance of optimizing bacterial IAA levels to avoid potential negative impacts on root structure, which are critical for water and nutrient uptake in crops. Ali et al. (2009) also noted significant variability in IAA production among bacterial strains, with differing effects on plant growth. While most strains promoted growth, others, such as *Bacillus* sp. AaH-1 and *Bacillus* sp. EpP-2, exhibited less pronounced or even neutral outcomes. This variability suggests that not all IAA-producing bacteria are equally beneficial, and their effects depend on host plant species and environmental conditions. Similarly, Barazani and Friedman (2000) reported that excessive IAA production, often driven by high concentrations of l-tryptophan, could inhibit primary root elongation. Castellanos Suarez et al. (2014) further demonstrated that *Micrococcus luteus*, an IAA-producing bacterium, acted as a deleterious rhizobacterium for *Arabidopsis thaliana*, reducing plant biomass and altering root architecture in a dose-dependent manner. In another study, rhizobacterial IAA accumulation significantly correlated with diminished root elongation in *Beta vulgaris* (sugar beet) following PGPB application (Loper and Schroth, 1986). Applying auxin (IAA)-producing pseudomonad PGPB similarly suppressed root elongation in *Arabidopsis thaliana* (Zamioudis et al., 2013). Furthermore, administering an IAA-deficient mutant of *Pseudomonas putida* GR12-2 to canola seedlings enhanced root elongation, demonstrating that elevated auxin concentrations inhibit root length (Patten and Glick, 2002). Such root growth inhibition may also be particularly detrimental under drought conditions, as stressed plants exhibit reduced auxin levels owing to boosted activity of the *GH3* gene, which conjugates active free auxins (Seo et al., 2009), and activation of IAA-oxidase (Li et al., 2014b). Consequently, drought-stressed plants may become more sensitive to microbial-produced auxins. Interestingly, the inhibitory effects of auxins on root elongation contrast with their stimulatory effects on shoot cell extension. Arteca and Arteca (2008) explained this phenomenon as dose-dependent, where increasing auxin concentrations stimulate the production of ethylene, a growth inhibitor (Glick, 2014). Elevated ethylene levels can exacerbate root elongation inhibition, potentially compromising drought tolerance. A study by Persello-Cartieaux et al. (2001) on *P. thivervalensis*, an IAA-producing strain, revealed that an inoculum density of 10^5^ CFU ml^−1^ induced favorable qualitative changes in colonized *Arabidopsis thaliana* plantlets, whereas densities exceeding 10^6^ CFU ml^−1^ caused irreversible damage. Another example is *Enterobacter* sp. I-3, which was found to inhibit the growth of radish and lettuce, likely due to its production of IAA in high concentrations. While IAA generally promotes plant growth, excessive levels can suppress seedling growth, as observed in radish and weeds (Park et al., 2015).

#### 5.1.2 Cytokinins

Cytokinins are a class of plant growth-regulating phytohormones that influence a wide range of developmental and physiological processes in plants. These processes include cell division, root elongation, seed germination, apical dominance, chloroplast differentiation, reproductive phase transition, flower and fruit development, leaf senescence, nutrient signaling, and interactions with pathogens (Akhtar et al., 2020; Arkhipova et al., 2007; Großkinsky et al., 2016). Beyond endogenous plant production, certain bacteria—both PGPB such as *Pseudomonas stutzeri, Stenotrophomonas maltophilia*, and *Pseudomonas putida*, as well as phytopathogens—synthesize cytokinins like zeatin, zeatin riboside, and isopentenyladenine (Kudoyarova et al., 2019; Orozco-Mosqueda et al., 2023; Patel and Saraf, 2017). The balance between cytokinins and auxin is a critical determinant of plant organogenesis and root architecture (Vacheron et al., 2013). While cytokinins directly stimulate shoot cell division and elongation, they often inhibit root growth, as seen in *Arabidopsis* inoculated with *Bacillus amyloliquefaciens* UCMB5113, where elevated cytokinin and auxin levels suppressed primary root elongation (Asari et al., 2017; Werner et al., 2010). Conversely, introducing high cytokinin-producing strains like *Bacillus subtilis* IB 22 into wheat and lettuce rhizospheres enhanced leaf area by promoting shoot growth (Arkhipova et al., 2005; Werner et al., 2003). However, cytokinin production by microbes is not universally beneficial. Some bacteria such as *Rhodococcus* spp. exploit cytokinins to induce hormonal imbalances, causing abnormal growth deformities like leafy galls in diverse host plants (Creason et al., 2014). The form of cytokinin also influences its effects: ribosylated cytokinins (e.g., zeatin riboside) are transported to shoots, promoting leaf growth without root inhibition, whereas non-ribosylated forms retained in roots may suppress root development (Kudoyarova et al., 2019). Thus, microbial cytokinin production can either enhance plant resilience or disrupt growth, underscoring the need for strain-specific optimization in agricultural applications.

#### 5.1.3 Gibberellins

Gibberellins (GAs) play a pivotal role in enhancing plant growth and productivity, with applications spanning agriculture and horticulture. Microbial gibberellins, particularly GA₃, are widely utilized to stimulate α-amylase activity in barley for malt production, boost grape yields in viticulture, and improve fruit size in citrus orchards (Cui et al., 2020). Additionally, GA₄/GA₇ mixtures reduce russeting in apples, induce flowering in conifers, and, when combined with auxins, enhance tomato quality and yield (Cui et al., 2020; Rouphael et al., 2021). PGPB-synthesized gibberellins can boost crop stem growth, alter the dormancy of germinating seeds, and enhance crop biomass and leaf and fruit senescence (Gupta and Chakrabarty, 2013; Kang et al., 2012; Keswani et al., 2022; Lee et al., 2015; Woo et al., 2023; Yamaguchi, 2008). Despite the benefits, the absence of regulatory mechanisms in microbial GA production could theoretically intensify resource competition between vegetative and reproductive growth (Cui et al., 2020; Zhang et al., 2022). For example, certain bacterial strains, such as *Enterobacter* sp I-3, can inhibit the gibberellin biosynthetic pathway, thereby affecting plant growth negatively (Radhakrishnan et al., 2016). These risks emphasize the necessity for strain-specific optimization to prevent adverse effects while utilizing GA-producing bacteria in agriculture. Additionally, the excessive or unregulated application of gibberellins—whether microbial or otherwise—may disrupt the balance of endogenous hormones, potentially leading to phytotoxicity or diminished crop quality. This underscores the importance of precise management to mitigate negative outcomes.

#### 5.1.4 Abscisic acid

Abscisic acid (ABA) is a critical phytohormone governing plant growth, development, and stress adaptation. It is generally associated with stress responses, including stomatal closure to reduce water loss and regulates seed dormancy, maturation, and germination, ensuring timely developmental transitions and preventing premature sprouting (Chen et al., 2020; Kavi Kishor et al., 2022). It also balances endogenous hormone levels and regulates growth metabolism, integrating internal and external signals to maintain homeostasis (Mo et al., 2024). Some PGPB can also produce ABA (Cohen et al., 2015, 2009; Kudoyarova et al., 2019; Pan et al., 2019; Shahzad et al., 2017). While ABA can protect plants under severe stress, it can also inhibit growth under moderate stress conditions by reducing photosynthesis and overall plant vigor (Kudoyarova et al., 2019).

### 5.2 Phytotoxic metabolites

Volatile Organic Compounds (VOCs) are low molecular weight (<300 Da), lipophilic molecules characterized by high vapor pressure and low boiling points, enabling rapid evaporation and diffusion through air and water (Bailly and Weisskopf, 2012; Montejano-Ramírez et al., 2024). These compounds are universally produced by organisms across kingdoms and serve critical roles in ecological interactions, including plant-microbe communication (Schulz and Dickschat, 2007). Microbial VOCs (mVOCs) from beneficial bacteria are well-documented for their ability to enhance plant growth, stress tolerance, and defense responses by modulating phytohormone pathways, including auxin, cytokinin, and ethylene signaling, and antimicrobial action (Montejano-Ramírez et al., 2024; Poveda, 2021; Ryu et al., 2004; Zhang et al., 2007). In growth promotion, Bacillus-derived 2,3-butanediol and acetoin increase *Arabidopsis thaliana* shoot biomass (Asari et al., 2016; Rath et al., 2018; Ryu et al., 2003) and lettuce root architecture (Fincheira et al., 2016). *Bacillus* spp. emit tetrahydrofuran-3-ol and 2-heptanone, which upregulate auxin and strigolactone pathways in *A. thaliana* and tomato (Jiang et al., 2019), while indole from *Proteus vulgaris* activates auxin, cytokinin, and brassinosteroid signaling (Bhattacharyya et al., 2015). Dimethyl disulfide from *Stenotrophomonas maltophilia* and *Pseudomonas stutzeri* promotes tomato growth (Rojas-Solís et al., 2018), and *B. subtilis* VOCs (e.g., albuterol) elevate gibberellins, auxins, and cytokinins while reducing ethylene (Tahir et al., 2017). *Pseudomonas fluorescens, Azospirillum brasilense*, and *P. putida* VOCs (e.g., 4-octylbutan-4-olide) enhance essential oil production in *Mentha piperita* (Santoro et al., 2016, 2011). However, high concentrations of ammonia or dimethyl disulfide from *Serratia odorifera* inhibit growth (Kai et al., 2010). For abiotic stress tolerance, *B. amyloliquefaciens* acetoin increases salicylic acid (SA) in *M. piperita*, improving salt resilience (Cappellari and Banchio, 2020). *Pseudomonas simiae* VOCs (phenol-2‑methoxy and stearic acid) reduce Na⁺ and upregulate peroxidase/catalase genes in soybean (Vaishnav et al., 2015, 2016). *B. subtilis* 2,3-butanediol modulates K⁺ transporters to confer salt tolerance (Zhang et al., 2008), while *Pseudomonas chlororaphis* emits the same VOC for SA-dependent drought resistance (Cho et al., 2008). In defense induction, *B. subtilis* acetoin primes SA-mediated resistance against *Pseudomonas syringae* in *A. thaliana* (Rudrappa et al., 2010). 2,3-Butanediol from *Bacillus* spp. and *P. chlororaphis* activates ethylene (ET)-signaling against *Erwinia carotovora* (Han et al., 2006; Ryu et al., 2004). *P. fluorescens* VOCs (3-nonene, 4-undecyne, and 1-undecene) induce systemic resistance while promoting growth (Cheng et al., 2016). α-Farnesene from *B. pumilus* and *B. amyloliquefaciens* boosts fruit defenses against *Peronophythora litchi* (Zheng et al., 2019). mVOCs also induce systemic resistance (ISR/SAR) against pathogens by activating defense genes like *PR1* and *PDF1.2* via salicylic acid (SA) and jasmonic acid (JA) pathways (Cho et al., 2008; Ryu et al., 2004). *B. subtilis* VOCs (benzaldehyde and nonanal) disrupt *Clavibacter michiganensis* via colony suppression and cellular abnormalities (Rajer et al., 2017). *B. amyloliquefaciens* emits 2-undecanone and 2-tridecanone, inhibiting motility and biofilm formation in *Ralstonia solanacearum* (Raza et al., 2016a, 2016b). *Pseudomonas putida* dimethyl trisulphide exhibits nematicidal activity against *Radopholus similis* (Agisha et al., 2019), while *P. donghuensis* VOCs (hydrogen cyanide and methyl thiocyanate) suppress oomycetes (Ossowicki et al., 2017). *Streptomyces alboflavus* dimethyl trisulfide inhibits aflatoxin production in *Aspergillus* spp. (Yang et al., 2019), and *B. amyloliquefaciens* 1,3-pentadiene reduces postharvest decay in cherries (Gotor-Vila et al., 2017).

Conversely, certain bacterial VOCs exhibit plant growth-inhibitory effects, particularly when strains like *Pseudomonas aeruginosa, Serratia plymuthica*, or *Chromobacterium violaceum* are cultured on nutrient-rich media such as LB, producing phytotoxic compounds like HCN, dimethyl disulfide (DMDS), and ammonia (NH_3_) (Blom et al., 2011; Rudrappa and Bais, 2008). The VOCs, such as HCN, dimethyl sulfide, and inorganic volatiles, have been inhibited plant growth by causing leaf necrosis or bleaching, as observed in studies involving *Bacillus mojavensis* RRC101 and other strains (Blom et al., 2011; Ryu et al., 2003; Vespermann et al., 2007). This bacterial phytotoxicity is attributed to the production of high levels of acetone/2-propanone, which may contribute to growth inhibition (Rath et al., 2018). It is known that HCN from cyanogenic bacteria also disrupts mitochondrial function and induces oxidative stress, leading to root inhibition or plant death in *Arabidopsis* and wheat (Åström and Gerhardson, 1989; Blom et al., 2011), while DMDS and NH_3_ from *Serratia* species suppress growth by altering phytohormone balance and photosynthetic efficiency (Kai and Piechulla, 2010). HCN, produced by certain bacterial strains, has been identified as a major factor responsible for volatile-mediated toxicity. HCN disrupts mitochondrial respiration by inhibiting cytochrome c oxidase, leading to impaired cellular function, while also inducing oxidative stress through the accumulation of reactive oxygen species (ROS) that damage plant tissues (Blom et al., 2011). In a study, Serratia plymuthica and Pseudomonas brassicacearum, isolated from the roots of *Jacobaea vulgaris*, significantly reduced root growth and seed germination of various plant species through direct root inoculation and volatile emissions (Liu et al., 2024). In another study, Song et al. (2021) identified pyrazine and 2,5-dimethylpyrazine as plant growth–inhibiting volatiles (PGIVs) emitted by *Bacillus amyloliquefaciens* GB03. These compounds significantly reduced plant growth at close distances (e.g., 7 cm) due to their ability to increase salicylic acid (SA) levels in plants, which suppressed growth when concentrations exceeded a certain threshold. *Arabidopsis* plants exposed to these volatiles exhibited 3.1- and 4.8-times higher SA content compared to controls, leading to growth inhibition. Additionally, exposure to synthetic 2,5-dimethylpyrazine caused extensive trypan blue staining in leaves, indicating cell death (Song et al., 2021). In natural ecosystems, allelopathic non-pathogenic bacteria, such as certain actinobacteria and pseudomonads, accumulate in the rhizosphere of allelopathic shrubs like *Adenostoma fasciculatum* and *Coridothymus capitatus*. These bacteria synergize with plant-derived phytochemicals to inhibit the germination and growth of nearby annuals, either directly via phytotoxins (e.g., geldanamycin and nigericin) or indirectly by suppressing essential symbionts like mycorrhizal fungi (Barazani and Friedman, 2001). In agricultural systems, continuous monocropping fosters yield decline due to deleterious rhizobacteria (DRB), such as *Pseudomonas fluorescens*, which thrive on repeated root exudates and release inhibitory metabolites. For instance, prolonged cultivation of barley, corn, or potatoes in the same soil leads to growth suppression without disease symptoms, attributed to DRB-produced compounds like hydrogen cyanide (Barazani and Friedman, 2001). The dual role of bacterial metabolites—promotion or inhibition—depends on bacterial strain, culture conditions, and plant developmental stage, underscoring their context-dependent ecological significance (Bailly and Weisskopf, 2012; Blom et al., 2011). This complexity highlights the need for further research to harness beneficial metabolites for sustainable agriculture while mitigating phytotoxic risks.

#### 5.3 Competition and antagonism with plant and indigenous microbes

The introduction of PGPB into soil systems often triggers intense competition for resources in the rhizosphere, which can paradoxically hinder plant growth despite the intended benefits. Root exudates, such as sugars and organic acids, attract fast-growing r-strategist bacteria (e.g., *Pseudomonas* and *Bacillus* species), which rapidly consume these energy-rich compounds and immobilize essential nutrients like N and P in their biomass, creating transient nutrient sinks (Fontaine et al., 2003; Henriksen and Breland, 1999; Kuzyakov et al., 2000; Paul, 2007). This competition is amplified by the "priming effect," where fresh organic matter stimulates microbial activity, further diverting nutrients away from plants (Bardgett et al., 2003; Fontaine et al., 2003). For instance, Dunn et al. (2006) demonstrated that microbial uptake of nitrogen—particularly in organic forms like glycine—exceeds plant assimilation in grassland soils, especially when microbial activity is enhanced by carbon inputs (e.g., glucose). This dynamic forces plants to expend additional energy on root exudate production with diminishing returns, exacerbating nutrient stress during critical growth stages (Liljeroth et al., 1994; Merckx et al., 1985). The ecological consequences extend beyond nutrient competition. Dominant r-strategists disrupt beneficial microbial alliances, such as mycorrhizal fungi, which are critical for nutrient mobilization (Raaijmakers et al., 2009). For example, *Acinetobacter calcoaceticus* P23 and *Pseudomonas fulva* Ps6, known to promote duckweed growth in nutrient-rich environments, inhibited *Lemna gibba* in nitrogen-poor wastewater due to fierce competition for limited N, while indigenous *Chryseobacterium* strains, with lower metabolic demands, enhanced growth under the same conditions (Khairina et al., 2021). This highlights the importance of tailoring PGPB selection to specific environmental contexts.

Antagonistic interactions further complicate inoculant efficacy. Native microbes often outcompete introduced strains through niche exclusion or inhibitory mechanisms. For instance, *Pythium irregular* suppresses *Pseudomonas fluorescens* Q72a-80 populations in wheat rhizospheres, while non-pathogenic *Fusarium* strains produce fusaric acid, blocking antibiotic synthesis in PGPB (Dutta and Podile, 2010). Even non-pathogenic PGPB can inadvertently harm plants under stress: certain *Pseudomonas* and *Bacillus* strains inhibit growth by monopolizing resources or secreting phytotoxic metabolites (Ishizawa et al., 2017). Co-inoculation strategies frequently backfire due to inter-strain competition. Felici et al. (2006) observed that combining *Bacillus subtilis* and *Azospirillum brasilense* distorted tomato root architecture, negating potential synergies. Similarly, multi-strain inoculants often provoke competitive exclusion, destabilizing both native communities and inoculant performance (Trabelsi and Mhamdi, 2013). Ultimately, the success of microbial inoculants hinges on balancing their competitive traits with ecological compatibility. Native microbial communities, adapted to local conditions, frequently resist introduced strains (Ravanbakhsh et al., 2018), underscoring the need for rigorous pre-testing and selection of PGPB that complement—rather than disrupt—indigenous microbiota. Addressing these challenges is critical to harnessing the full potential of microbial inoculants while safeguarding soil health and plant productivity.

## 6 Negative impacts on soil ecosystems

The inoculation of bacteria into soil can have significant negative effects on both the native microbial community and the introduced bacteria themselves. According to the literature, PGPB primarily affect soil ecosystems through mechanisms such as disruption of soil microbial diversity, altered nutrient cycling and soil chemistry, and potential for pathogenicity and horizontal gene transfer (Fig. 3 and Table 2)

### 6.1 Disruption of soil microbial diversity

Microbial inoculation, a deliberate biotic disturbance, introduces non-native species into soil ecosystems, potentially disrupting resident microbial communities through competitive, mutualistic, or antagonistic interactions (Liu et al., 2022, 2023). While the goal is to enhance plant performance, this practice risks unintended consequences: native microbial diversity—a critical driver of ecosystem functions like nutrient cycling and plant productivity—may decline, indirectly compromising agricultural outcomes (Bardgett and Van Der Putten, 2014; Cornell et al., 2021; Van Der Heijden et al., 1998). PGPB, acting as invaders, can perturb established microbial niches, leading to three possible outcomes—stable establishment with lasting community shifts, resilience-driven restoration of pre-invasion conditions, or transient disruption followed by recovery (Kinnunen et al., 2018; Mallon et al., 2015, 2018; Mawarda et al., 2020). Even transient inoculants may leave enduring “legacy effects,” defined as persistent ecosystem changes outlasting the inoculant’s presence (Cuddington, 2011; Simard et al., 1997). These legacies arise through direct functional traits (e.g., exopolysaccharide production improving soil water retention), plant-mediated effects (e.g., phytohormone-driven root modifications), or microbial community restructuring via competitive interactions (Gamalero et al., 2020; Gamalero and Glick, 2019; Hu et al., 2021; Liu et al., 2022). Bacterial communities are more vulnerable to inoculation-induced shifts than fungal communities, likely due to fungal structural resilience, while multi-strain consortia provoke stronger disruptions than single strains (Cornell et al., 2021). Although inoculant populations often decline over time, legacy effects may persist, either reducing biodiversity through competitive exclusion or enhancing it via suppression of dominant taxa (Janoušková et al., 2017; Mawarda et al., 2020; Vuolo et al., 2022). Research interest in these non-target effects has surged, with annual publications rising from 445 (2019) to 728 (2022), reflecting growing recognition of the complex trade-offs between agricultural productivity and ecological stability in inoculated agroecosystems (Cornell et al., 2021).

Studies by Thomas and Sekhar (2016) demonstrated that *Pseudomonas aeruginosa* inoculation caused an immediate but short-lived suppression of native bacteria, reducing colony-forming units (CFU) significantly, followed by native community recovery within a week. This resilience was attributed to antagonism from native microbiota, as the inoculant itself declined by 99 % in non-sterile soil but thrived in sterile conditions. Similarly, Schreiter et al. (2014) observed soil-type-dependent disruptions in lettuce rhizospheres with *Pseudomonas jessenii*, while Matos et al. (2005) noted that higher native microbial diversity in wheat rhizospheres reduced *P. aeruginosa* invasiveness, emphasizing the role of biodiversity in ecosystem resistance. These dynamics are driven by resource competition and antagonism, as seen in wheat roots, where Conn and Franco (2004) reported a 47 % reduction in actinobacterial endophyte diversity after applying the commercial inoculant NutriLife 4/20, which outcompeted native genera like *Streptomyces* and *Microbispora*, impairing plant-microbe symbiosis. Similarly, Robleto et al. (1998) found that *Rhizobium etli* producing trifolitoxin suppressed α-proteobacteria, risking nitrogen cycling functions, while Van Veen et al. (1997) highlighted broader challenges like predation and competition faced by introduced strains. The transient nature of disruptions is exemplified by Gao et al. (2012)*,* where *Pseudomonas fluorescens* 2P24 temporarily altered cucumber rhizosphere fungal communities, and Baudoin et al. (2009)*,* where *Azospirillum lipoferum* CRT1 increased genetic variability in maize rhizobacteria, destabilizing microbial predictability. However, legacy effects—persistent ecological changes—can arise, such as plasmid transfer from genetically modified *Sinorhizobium meliloti* to native strains (Morrissey et al., 2002) or reduced *Pseudomonas* populations in broccoli endospheres (Gadhave et al., 2018), compromising disease suppression. Physical disturbances also exacerbate these impacts: Cornell et al. (2021) reported that 89 % of inoculation studies in disturbed soils (e.g., tilled fields) saw reduced bacterial diversity, with 88 % linking diversity loss to stunted plant growth, underscoring the critical role of native microbes in nutrient cycling and stress resilience (Bardgett and Van Der Putten, 2014). Multi-strain inoculants amplified risks, as seen in cucumber, where a consortium (*Ensifer, Acinetobacter,* and *Flavobacterium*) reduced Actinobacteria and Firmicutes (Wang et al., 2018b), and in maize, where *Azospirillum argentinense* Az39 lowered bacterial evenness (Coniglio et al., 2022).

Functional disruptions are further illustrated by Sun et al. (2009)*,* where rhizobial inoculation in alfalfa shifted ammonia-oxidizing bacteria dominance (*Nitrosomonas* to *Nitrosospira*), altering nitrogen cycling, and Florio et al. (2017)*,* where *A. lipoferum* CRT1 reduced denitrifier abundance in carbon-limited soils. Even in extreme environments, such as acidic mine tailings, De-Bashan et al. (2010) observed structural community shifts post-inoculation, highlighting ecological sensitivity. While some studies, like Schwieger and Tebbe (2000) and Van Dillewijn et al. (2002)*,* noted proteobacterial redistributions (e.g., increased Alphaproteobacteria and reduced Gammaproteobacteria) with uncertain long-term effects, others warned of horizontal gene transfer risks (Morrissey et al., 2002) or reduced colonization of beneficial taxa (Trabelsi and Mhamdi, 2013). These findings collectively emphasize the dual role of microbial inoculants: while offering agricultural benefits, they risk destabilizing soil ecosystems through competition, antagonism, and legacy effects. Liu et al. (2023) and Liu et al. (2022) stress the need to prioritize microbial community dynamics in research, particularly in understanding inoculant-driven restructuring of rare taxa and root exudate profiles. To mitigate risks, pre-screening inoculants for narrow-spectrum activity (Thomas and Sekhar, 2016), avoiding overly competitive multi-strain consortia (Cornell et al., 2021), and selecting strains that complement native microbiota (Conn and Franco, 2004) are critical. Sustainable deployment hinges on balancing short-term agronomic gains with long-term ecological stability, necessitating policies that prioritize biodiversity conservation and rigorous monitoring of non-target effects (Liu et al., 2023). Most of the included studies primarily focused on functional outcomes, such as plant growth and pollutant removal, rather than on microbial community dynamics. There is a need for future research to address this gap.

### 6.2 Altered nutrient cycling and soil chemistry

The introduction of non-native bacteria into soil ecosystems can disrupt critical nutrient cycles and undermine soil health by displacing native microbial taxa that perform essential ecosystem functions. Microbial inoculants, though often deployed to enhance agricultural productivity, risk destabilizing soil nutrient dynamics, particularly N cycling, and organic matter decomposition. For instance, inoculating cucumber with a bacterial consortium (*Ensifer, Acinetobacter*, and *Flavobacterium*) reduced populations of Actinobacteria and Firmicutes compared to controls (Wang et al., 2018b). These groups are vital for organic matter decomposition and antibiotic production, suggesting that their decline could impair soil health and resilience. Similarly, *Bacillus amyloliquefaciens* inoculation in broccoli decreased beneficial *Pseudomonas* spp. populations in the plant endosphere (Gadhave et al., 2018), potentially compromising plant growth promotion and pathogen suppression services provided by these Gammaproteobacteria. Disruptions to nitrogen cycling are particularly evident. Inoculating alfalfa with *Sinorhizobium meliloti* L33 increased Alphaproteobacteria but reduced Gammaproteobacteria (Schwieger and Tebbe, 2000). including keystone taxa like *Pseudomonas*. A related strain, *S. meliloti* M401/M403, altered γ-proteobacterial persistence in alfalfa rhizospheres (Van Dillewijn et al., 2002), potentially hindering organic matter decomposition and nitrogen fixation. Rhizobial inoculants in alfalfa also shifted ammonia-oxidizing bacterial dominance from *Nitrosomonas* to *Nitrosospira* (Sun et al., 2009), which may destabilize nitrification processes. Furthermore, *Azospirillum lipoferum* CRT1 inoculation reduced denitrifier abundance in carbon-rich soils by intensifying competition for nitrate (Florio et al., 2017), highlighting how carbon availability modulates inoculant impacts on N cycling. Inoculants may also erode microbial diversity critical for ecosystem stability. For example, a *Rhizobium etli* strain engineered to produce trifolitoxin suppressed diversity-sensitive α-proteobacteria in bean rhizospheres (Robleto et al., 1998), risking the loss of native taxa involved in nutrient cycling. A seminal study by Philippot et al. (2013) also demonstrated that even modest reductions in microbial diversity—achieved through serial dilution of soil communities—severely impaired denitrification, a key process converting nitrate to inert nitrogen gas. A 75 % reduction in denitrifier diversity caused a 4–5 fold decline in denitrification rates, underscoring the limited functional redundancy—*multiple taxa performing identical ecosystem functions*—in microbial guilds. This finding implies that introduced bacteria, which may outcompete native species, could similarly degrade nitrogen cycling by eroding the diversity and functional capacity of indigenous microbial communities. Such disruptions extend beyond nitrogen. Native microbes drive processes like phosphate solubilization, organic matter decomposition, and disease suppression. Their loss reduces the resilience and adaptive capacity of soil ecosystems (Kennedy, 1999), leaving them vulnerable to environmental stressors. For example, Trabelsi and Mhamdi (2013) documented how inoculants targeting specific plant pathogens inadvertently suppressed beneficial taxa involved in nutrient mineralization, leading to imbalances in carbon and nitrogen pools. These shifts can cascade into broader ecological consequences, such as reduced soil organic matter stability or increased greenhouse gas emissions. The decline in functional stability also compromises the soil’s ability to buffer against disturbances. Microbial diversity acts as an insurance policy; even if some species are lost, others can maintain ecosystem processes. However, inoculants that displace keystone species—such as nitrifiers, denitrifiers, or mycorrhizal symbionts—create “functional gaps” that degrade soil fertility over time. This is particularly problematic in agricultural systems, where repeated inoculation may exacerbate nutrient imbalances, requiring increased synthetic fertilizer use to compensate. Ultimately, the unintended consequences of microbial inoculants on nutrient cycling highlight the need for precision in their application. Preserving native microbial diversity is critical to sustaining soil health, and inoculant strategies must prioritize compatibility with indigenous communities to avoid destabilizing the very processes they aim to enhance.

### 6.3 Potential for pathogenicity and horizontal gene transfer

Horizontal gene transfer (HGT)—a key evolutionary mechanism enabling bacteria to acquire genetic material from unrelated organisms via conjugation, transduction, or transformation (Keeling and Palmer, 2008; Redondo-Salvo et al., 2020)—poses significant risks in agricultural systems where rhizosphere biofilms, root exudates, agrochemical stressors (e.g., glyphosate), and intensive farming practices concentrate microbial density and extracellular DNA, accelerating genetic exchange (Deng et al., 2017; Ghaly and Gillings, 2018; Ghaly et al., 2024; Kurenbach et al., 2015). Integrons, prevalent in plant-associated orders like *Pseudomonadales* and *Burkholderiales*, act as HGT hotspots by capturing gene cassettes that confer virulence or plant-growth traits across phylogenetic boundaries (Mahdi et al., 2022; Stritzler et al., 2018).

Critically, HGT is not merely a risk factor but an evolutionary accelerator that enables rapid niche adaptation. Environmental stressors (e.g., pesticides and heavy metals) can upregulate competence genes, increasing DNA uptake rates by 3–5 fold and facilitating the emergence of hybrid strains with unpredictable impacts on soil ecosystems (Ghaly and Gillings, 2018; Kurenbach et al., 2015). Studies demonstrate that bacterial genomes can acquire new genetic material through HGT, significantly altering their relationship with plants. For example, *Rhodococcus* bacteria can shift from being beneficial plant growth-promoting symbionts to phytopathogens upon acquiring a virulence plasmid (Savory et al., 2017). This evolutionary transition highlights how HGT can rapidly generate new pathogenic lineages, illustrating that beneficial bacteria can become harmful through the acquisition of pathogenic genes. PGPB may acquire pathogenicity genes through: (1) conjugative plasmids/ICEs transferring phytotoxin operons (e.g., *Pseudomonas syringae* coronatine genes) (Cuppels and Ainsworth, 1995; Vladimirov et al., 2015), (2) phage-mediated transduction of type III secretion systems (*hrp* genes) (Deng et al., 2017; Lee et al., 2022), or (3) stress-induced transformation enabling uptake of pathogen-derived DNA (e.g., pectate lyases) (Hernández-Morales et al., 2009; Johnsborg et al., 2007). Such transfers can trigger functional switches—exemplified by the *Lap* cluster transfer from beneficial *Pseudomonas fluorescens* to phytopathogenic *Erwinia* (Ghaly et al., 2024)—reprogramming PGPB to disrupt root development, suppress immunity, or cause disease symptoms (Ghaly and Gillings, 2022; Loiko and Islam, 2024; Singh et al., 2023). The persistence of genetically modified microbes (e.g., *Sinorhizobium meliloti* plasmids) (Morrissey et al., 2002) and HGT of virulence genes (e.g., opine biosynthesis and coronatine) to non-target strains raises concerns about mutualists converting into latent pathogens, undermining microbial inoculant reliability and reducing crop yields (Ghaly and Gillings, 2022; Moura de Sousa et al., 2023; Rajabal et al., 2024). Thus, elucidating HGT pathways—driven by microbial proximity, selective pressures, and bacterial DNA integration capacity—is critical for monitoring gene flow and designing safer agricultural microbiome management strategies.

#### 6.3.1 MGEs in commercial strains and their role in ARGs transfer

PGPB used as biofertilizers frequently harbor antibiotic resistance genes (ARGs) that can be transferred to other bacteria through HGT mechanisms. These ARGs are often located on mobile genetic elements (MGEs) such as plasmids, transposons, and integrative conjugative elements (Mahdi et al., 2022). The large-scale application of such bacteria in agricultural systems may serve as a source of ARG invasion which can potentially enter the food chain, presenting risks to ecosystem and public health (Mahdi et al., 2022). Research has shown that many *Bacillus* species, commonly used as bio-fungicides and biofertilizers, are multi-drug resistant (MDR) and carry various antimicrobial resistance genes including *VmiR, ImrB, tetL, mphK, ant-6, penp*, and *bla OXA* (Wash et al., 2022). The multiple antimicrobial resistance (MAR) index of these *Bacillus* strains has been found to be higher than the critical value (0.2), indicating significant resistance to multiple antibiotic classes like β-lactams, macrolides, sulfonamides, tetracyclines, and aminoglycosides (Wash et al., 2022). The horizontal transfer of ARGs, between introduced biofertilizers and indigenous soil microorganisms occurs through several mechanisms. Conjugation involves direct cell-to-cell contact, allowing for plasmid transfer; for instance, studies have demonstrated the successful transfer of genes like *nifH2* and *nifH3* from *Azotobacter chroococcum* to *Bacillus megaterium* (Talabani et al., 2019). Transformation entails the uptake of extracellular DNA from the environment, exemplified by the successful transfer of *nodD2* and *nodD3* genes from *Rhizobium leguminosarum* to *Bacillus megaterium* (Talabani et al., 2019). Additionally, transduction, mediated by bacteriophages, facilitates the transfer of genetic material between bacteria. Recent data highlight the significance of transduction in the spread of resistance, particularly in wastewater treatment plants, which can impact agricultural soils when treated wastewater is used for irrigation (Lood et al., 2017). The rhizosphere serves as a hotspot for horizontal gene transfer due to its high microbial density and activity. When bacteria containing ARGs are introduced as biofertilizers, several risk factors emerge. First, the application of animal manure, which often contains antibiotic-resistant bacteria (ARBs) and ARGs, leads to increased ARG abundance in the soil-water system, facilitating the propagation and dissemination of resistance genes (Checcucci et al., 2020). Additionally, ARGs can be transferred to opportunistic human pathogens (OHPs) residing in the phyllosphere of leafy vegetables, posing significant risks to food safety and human health (Lin et al., 2023). For example, phagotrophic protists have been shown to preserve antibiotic-resistant OHPs in vegetable phyllospheres (Lin et al., 2023). Furthermore, ARGs located on MGEs can persist in the environment even after the original bacterial host is eliminated, allowing for future transfer events (Checcucci et al., 2020; Lood et al., 2017). These studies show that the presence of MGEs carrying ARGs can pose significant risks for the spread of antibiotic resistance in agricultural environments. Comprehensive screening procedures, regulatory frameworks, and innovative biotechnological approaches are needed to maximize the benefits of biofertilizers while minimizing the risks associated with horizontal gene transfer of ARGs.

## 7 Factors influencing the dual behavior of PGPB

The interaction between PGPB and host plants is a dynamic and interdependent relationship shaped by biotic and abiotic factors in the rhizosphere. This interplay involves plant and bacterial genotypes, soil geochemistry, microbial community dynamics, and environmental stressors, all of which determine whether PGPB exhibit growth-promoting or inhibitory effects (Ambrosini et al., 2016; Dutta and Podile, 2010). Optimizing these interactions is critical for maximizing agricultural benefits while minimizing risks to plant health.

### 7.1 Environmental conditions and soil type

Environmental conditions, including soil type, play a pivotal role in driving dual behavioral shifts in inoculated beneficial bacteria, often transforming their growth-promoting effects into detrimental outcomes for plants (Liu et al., 2023). Soil properties such as pH, organic matter (OM) content, and nutrient availability directly modulate bacterial functionality: neutral soils (pH 6–7) enhance microbial activity and symbiosis, whereas acidic conditions (pH < 5.5) reduce nutrient availability and increase phytotoxic metals like aluminum, suppressing rhizobacterial growth and antibiotic production (Liu et al., 2023; Nehl et al., 1997). Low OM and clay content amplify auxin (IAA) diffusion, which under drought stress can overstimulate root inhibition instead of promoting growth, as seen in maize exposed to *Arthrobacter* sp. under fluctuating soil moisture (Araújo et al., 2023; Kudoyarova et al., 2019; Persello‐Cartieaux et al., 2003). Similarly, nutrient imbalances, such as high phosphate levels, repress antibiotic synthesis in *Pseudomonas* and *Bacillus* strains, diminishing their biocontrol efficacy and leaving plants vulnerable to pathogens (Liu et al., 2023). Agrochemical inputs exacerbate these dynamics—nitrogen fertilizers disrupt ammonia-oxidizing bacteria (Enwall et al., 2005; Hallin et al., 2009), while fungicides suppress non-target microbes, destabilizing the cooperative networks that inoculated strains rely on for survival (De Vries et al., 2012; Wainwright and Wainwright, 1999). Environmental stressors like drought, extreme temperatures, or high humidity further tip the balance: drought-stressed plants allocate fewer resources to sustain symbionts, rendering beneficial bacteria parasitic (Cheng et al., 2019; Velásquez et al., 2018), while humidity-driven pathogen proliferation (e.g., *Pseudomonas syringae* or *Escherichia coli*) can exploit weakened plant defenses, turning inoculated strains into inadvertent enablers of disease (Hao et al., 2024; Ishizawa et al., 2017; Morris et al., 2017; Moyne et al., 2020). Even root exudates, which typically attract beneficial microbes, can backfire under stress—organic acids acidify the rhizosphere, inhibiting PGPR (Liu et al., 2023), while fusaric acid from non-pathogenic *Fusarium* suppresses bacterial antibiotic production (Liu et al., 2023). In non-sterile soils, competition with native microbes and predation by protozoa further constrain inoculant success, as seen in *Pseudomonas fluorescens* populations outcompeted by *Pythium* in wheat rhizospheres (Ambrosini et al., 2016; Liu et al., 2023). These dualities underscore the fragile equilibrium of plant-microbe interactions: while 92 % of inoculations show positive outcomes, 7.5 % of cases demonstrate inhibitory effects, where the same bacterial strain promotes growth in controlled settings but fails or harms plants in nutrient-poor, acidic, or dynamically stressed environments (Dadzie et al., 2023). Thus, environmental context dictates microbial behavior, emphasizing the need for tailored inoculation strategies that account for soil type, climatic stressors, and agrochemical regimes to mitigate unintended negative consequences (Ambrosini et al., 2016; Van Elsas et al., 2012).

### 7.2 Plant genotype and host-specific interactions

The dual effects of PGPB are profoundly shaped by plant genotype and host-specific interactions, with outcomes ranging from mutualism to antagonism due to mechanistic differences in plant-bacterial signaling and stress responses. Variability in inoculation outcomes—from negligible shifts to significant microbial community alterations—stems not only from experimental variables (inoculant concentration, strain, host species/genotype, plant age, soil type) (Cornell et al., 2021; Vuolo et al., 2022) but also fundamental molecular mismatches. Crucially, plant genotype determines sensitivity to bacterial metabolites like IAA through differential expression of auxin receptors (e.g., TIR1/AFB) and transporters (PINs/PGPs), creating species-specific dose-response curves: low IAA stimulates root elongation via cell wall acidification and expansion, while excessive IAA—produced by strains like *Enterobacter taylorae* and *Pseudomonas putida*—triggers ethylene biosynthesis *via* ACC synthase upregulation, leading to root inhibition, leaf senescence, and suppressed shoot growth (Gamalero and Glick, 2015; Sarwar and Kremer, 1995; Taiz et al., 2015; Xie et al., 1996). Strains secreting supraoptimal IAA (e.g., *Micrococcus luteus* at 195.1 ± 0.2 μM or *Streptoverticillium* sp. averaging 76.6 μM) inhibit lettuce roots by overwhelming these regulatory pathways, unlike moderate-IAA producers (16.4 μM) (Barazani and Friedman, 1999). Host-specificity further arises from genetic differences in plant immune perception: bacterial strains beneficial to tomato (*Lycopersicon esculentum*) or radish (*Raphanus sativus*) may activate defense responses (e.g., MAPK cascades, ROS bursts) in *Arabidopsis thaliana*, causing antagonism (Castellanos Suarez et al., 2014). Plants also actively modulate bacterial behavior by limiting tryptophan availability (IAA precursor) through root exudate composition—*Arabidopsis* reduces bacterial IAA synthesis by 43 % *via* this mechanism (Persello‐Cartieaux et al., 2003; Puga-Freitas et al., 2012). Similarly, *Bacillus megaterium* ORE8 and *Pantoea* sp. ORTB2 promote onion growth but inhibit pepper due to divergent phytohormone cross-talk in Solanaceae vs. Alliaceae (Samayoa et al., 2020). Antagonistic strains like *Serratia plymuthica* and *Pseudomonas brassicacearum* exploit host-specific vulnerabilities in weed rhizospheres (e.g., receptor affinity for toxins), suppressing germination in grasses/legumes but not crops (Liu et al., 2024). While 92 % of inoculations enhance growth, 7.5 % cause inhibition (Dadzie et al., 2023)—a duality exemplified by volatile organic compounds (VOCs), where strain-specific metabolite blends activate distinct plant signaling pathways (e.g., jasmonate vs. ethylene) depending on developmental stage and environment (Bailly and Weisskopf, 2012; Blom et al., 2011). Environmental stressors amplify mismatches: nutrient-poor sandy soils exacerbate IAA-mediated ethylene stress, while high inoculum densities saturate plant regulatory capacity, explaining *Pseudomonas putida*’s stronger dicot inhibition (Barazani and Friedman, 2001). Even commercial applications reflect this: bialaphos (derived from *Streptomyces hygroscopicus*) targets broadleaf weeds *via* genetically encoded glutamine synthetase inhibition, illustrating how allelopathy exploits host biochemical differences (Barazani and Friedman, 2001). Thus, PGPB effects hinge on molecular compatibility between bacterial effectors and plant genetic architecture, necessitating mechanistic screening to optimize strain-host pairings.

### 7.3 Physiological trade-offs as determinants of PGPB outcomes

The "Defense-Growth Trade-Off" is a fundamental concept in plant biology that describes the competition for finite resources between two critical processes: defense and growth (He et al., 2022). Defense involves the production of compounds and mechanisms that enable plants to resist pathogens, herbivores, and abiotic stresses. In contrast, growth focuses on the allocation of energy and nutrients to essential processes such as photosynthesis, root and shoot development, and reproduction. This trade-off highlights the challenge plants face in balancing their investment in defensive strategies with their need to grow and reproduce effectively. While beneficial bacteria such as *Pseudomonas, Bacillus*, and *Streptomyces* enhance plant defenses via induced systemic resistance (ISR), this protection often comes at the expense of plant growth and productivity. ISR, mediated by jasmonic acid (JA) and ethylene (ET) pathways, primes plants for rapid defense responses against pathogens but triggers significant metabolic trade-offs (Gozzo and Faoro, 2013; Vallad and Goodman, 2004). For instance, ISR activation redirects nitrogen and energy resources toward synthesizing defense compounds like pathogenesis-related (PR) proteins (e.g., chitinases) and phytoalexins, which can consume up to 10 % of a plant’s soluble leaf proteins under stress (Heil and Baldwin, 2002; Heil and Bostock, 2002). This reallocation reduces the resources available for growth processes such as root elongation, biomass accumulation, and reproductive development. In wheat, nitrogen-deficient plants treated with salicylic acid (SA) mimics—which mimic ISR signaling—exhibit stunted growth and reduced fitness, illustrating the growth-defense trade-off under resource-limited conditions (Heil et al., 2000). In another study, Song et al. (2021) also showed that high volatile concentrations suppress plant growth and promote immunity, aligning with the concept of the "defense-growth trade-off." Furthermore, ISR signaling pathways antagonize growth-promoting hormonal networks. JA/ET-mediated ISR suppresses auxin and gibberellin signaling, critical drivers of cell expansion and division (Walters and Heil, 2007). Transgenic plants with constitutively activated ISR frequently display dwarfed phenotypes, underscoring the incompatibility of sustained defense activation with normal development (Gaffney et al., 1993). Additionally, the metabolic burden of ISR leaves plants vulnerable to secondary stressors, such as opportunistic pathogens or abiotic stresses, further compounding growth inhibition (Gozzo and Faoro, 2013).

### 7.4 Evolutionary dynamics and adaptive shifts over time

The dualistic behavior of PGPB is further complicated by their capacity for rapid evolution and environmental adaptation. Under selective pressures—such as nutrient competition, host immune responses, or abiotic stressors—PGPB populations can undergo phenotypic and genotypic shifts that alter their functional outcomes. Horizontal gene transfer (HGT), plasmid acquisition, and mutations enable strains to acquire traits like enhanced virulence, antibiotic resistance, or altered metabolite production within short timeframes (Ghaly et al., 2024; Morrissey et al., 2002). For instance, commensal *Pseudomonas* strains can evolve pathogenic behaviors via HGT of phytotoxin-encoding plasmids (e.g., coronatine synthesis genes) in response to host oxidative stress (Hernández-Morales et al., 2009; Loiko and Islam, 2024). Similarly, repeated inoculation practices may select for "cheater" strains that exploit plant resources without providing mutualistic benefits, ultimately destabilizing plant-microbe symbioses (Kemen, 2014). These adaptive processes underscore the fluidity of PGPB-plant interactions, where initially beneficial inoculants may transition toward commensalism or antagonism over time.

### 7.5 Interkingdom signaling and plant immune modulation

Plant immune systems dynamically regulate interactions with PGPB through molecular recognition and signaling cascades. Pathogen-associated molecular patterns (PAMPs) from bacteria trigger MAMP-Triggered Immunity (MTI) via receptors such as FLS2 and EFR, leading to ROS bursts, callose deposition, and defense gene expression (Nishad et al., 2020; Ravanbakhsh et al., 2018). Beneficial PGPB have evolved mechanisms to evade or suppress MTI, including the secretion of effector proteins like AvrE from *Pseudomonas simiae*, which inhibits callose synthase and suppresses immune activation (Smith et al., 2015; Teixeira et al., 2019). Additionally, exopolysaccharides (EPS) from *Bacillus subtilis* biofilms can mask PAMPs, reducing ROS production by 60 % (Yang et al., 2024), while VOCs such as dimethyl disulfide from *Pseudomonas fluorescens* prime jasmonate signaling, balancing growth and defense responses (Phour et al., 2020; Yang et al., 2024). Plant-derived signals also shape these interactions; flavonoids recruit symbionts, activating nod genes in rhizobia while inhibiting the virulence of pathogens like *Agrobacterium* (Smith et al., 2015; Tadrosova et al., 2025). Furthermore, strigolactones attract mycorrhizal fungi but can suppress *Rhizobium* nodulation in phosphate-rich soils (Smith et al., 2015). This intricate molecular dialogue determines whether PGPB act as mutualists or stressors, emphasizing that the plant immune status is a critical factor in dictating microbial compatibility (Tadrosova et al., 2025; Teixeira et al., 2019).

## 8 Strategies for mitigating and managing adverse effects

### 8.1 Selection of safe and effective PGPB strains

The efficacy of PGPB in field conditions hinges on overcoming challenges posed by native soil microbial communities, which often outcompete introduced strains. Rigorous multi-stage screening—spanning lab, greenhouse, and field trials—is critical to identify strains with biofilm-forming abilities and stress-specific adaptations (e.g., salinity, heavy metal, and drought), enhancing root colonization and environmental resilience (Ajijah et al., 2023; Kumari et al., 2023). Prioritizing locally adapted strains or those pre-acclimated to regional abiotic stresses improves survival, as seen with *Glomus fasciculatum*-enhanced tomato root colonization attracting Pseudomonas fluorescens (Dutta and Podile, 2010). However, even robust strains may exhibit dual roles: *Burkholderia ambifaria* MCI 7 promoted maize growth as a seed treatment but suppressed it when soil-incorporated, underscoring application-dependent outcomes (Ciccillo et al., 2002). Similarly, *Enterobacter* sp. I-3 inhibited radish growth via excessive indole-3-acetic acid (IAA) production, highlighting the necessity for crop-specific strain screening (Park et al., 2015; Samayoa et al., 2020).

### 8.2 Optimizing application methods and dosages

Adapting inoculation strategies to crop growth stages and environmental conditions is paramount. Seed coatings, for instance, concentrate microbial activity near roots, whereas soil drenching ensures broader distribution but risks phytotoxicity (Esitken et al., 2010). Advanced formulations—such as encapsulation in alginate beads—protect PGPB from desiccation and UV radiation, enhancing field viability (Li et al., 2024). Meta-analyses reveal that short-term experiments (<1 month) overestimate inoculant efficacy, as effects diminish over time (Azarbad and Junker, 2024). For instance, *Bacillus mojavensis* RRC101 promoted *Arabidopsis* growth in sterile media but caused phytotoxicity on nutrient agar due to volatile organic compound shifts (Rath et al., 2018). Thus, field validation is essential to avoid misleading lab-based conclusions.

### 8.3 Integrated soil and plant health management

Soil properties profoundly influence inoculant success. Neutral pH (6–7), moderate organic matter, and balanced nutrient levels optimize microbial activity, while excessive N or P reduces reliance on PGPB (Azarbad and Junker, 2024; Liu et al., 2023). For example, liming acidic soils improved *Pseudomonas* survival by alleviating aluminum toxicity. Integrating PGPB with organic amendments (e.g., compost and biochar) enhances carbon availability, fostering synergistic interactions with mycorrhizal fungi for nutrient mineralization (Egamberdieva et al., 2018; Hammer et al., 2015; Jiang et al., 2021). However, non-native inoculants like *Streptomyces albidoflavus* disrupted wheat endophytic actinobacteria, reducing diversity by 48 % (Conn and Franco, 2004), emphasizing the need for indigenous consortia. One approach to mitigate the risks associated with ARG transfer from biofertilizers is the pre-treatment of organic amendments. Chemical and bio-sanitizing treatments can effectively reduce antibiotic loads and levels of antibiotic-resistant bacteria (ARBs) in organic amendments, such as animal manure, before their application in agriculture. This pre-treatment helps minimize the potential for ARG dissemination in the soil-plant system (Checcucci et al., 2020).

### 8.4 Mitigating ecological risks through consortia design

Multi-strain consortia with complementary functions (e.g., nitrogen fixation + phosphate solubilization) outperform single strains by enhancing adaptability and reducing competition. Co-inoculating *Bradyrhizobium* sp. and *Leclercia adecarboxylata* improved soybean nodulation and yield by 30 % (Kumawat et al., 2019). However, consortia must avoid ecological disruption: dual rhizobial inoculation (*Rhizobium gallicum* + *Ensifer meliloti*) unpredictably altered soil microbiota (Trabelsi et al., 2012). Quorum-sensing-mediated interactions and stress-resistant biofilms improve consortium resilience, but long-term ecological impacts remain poorly understood (Santoyo et al., 2021). The synthetic biology techniques can be also utilized to create engineered strains with a reduced potential for horizontal gene transfer or the removal of resistance genes while preserving beneficial traits (Ke et al., 2021). For instance, studies have demonstrated that in certain *Burkholderia* species, the removal of the third chromosomal replicon (over 1 Mb of DNA) does not affect their root colonization abilities on *Arabidopsis thaliana* (Vidal-Quist et al., 2014). This finding suggests the potential for developing virulence-attenuated strains suitable for biotechnological applications, enhancing safety while maintaining efficacy in agricultural settings.

### 8.5 Addressing phytotoxicity and hormonal imbalances

Phytotoxic VOCs like hydrogen cyanide from *Pseudomonas aeruginosa* PAO1 disrupt mitochondrial function and induce oxidative stress, necessitating screening for cyanogenic activity (Blom et al., 2011). Breeding plants with antioxidant defenses (e.g., overexpressing AOX1a) or using non-cyanogenic strains mitigates toxicity. Hormonal conflicts, such as cytokinin-ABA trade-offs, require strain-specific testing to balance growth and stress responses (Rabari et al., 2023).

### 8.6 Standardization and long-term monitoring

**Fig. 4:**
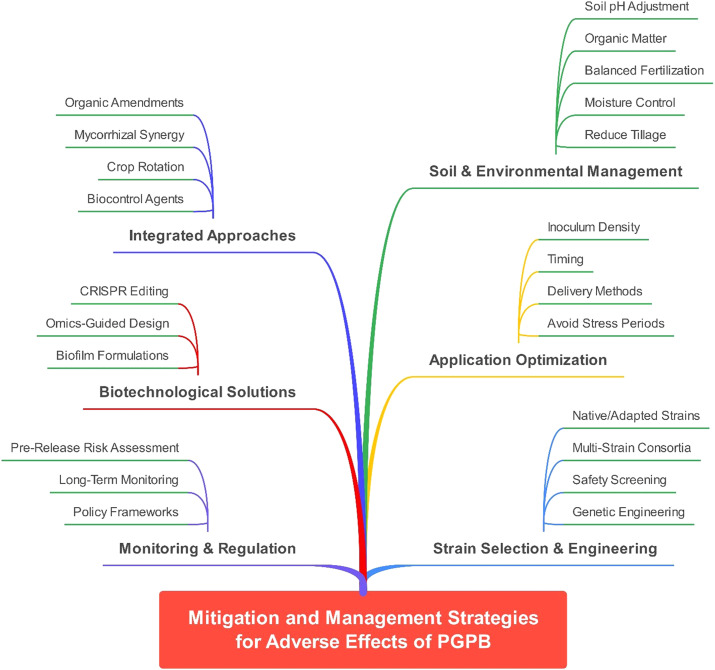
Strategies for mitigating and managing adverse effects of introduced Bacteria into soil and plants. This figure outlines evidence-based strategies to balance the benefits of plant growth-promoting bacteria (PGPB) with ecological safety, addressing risks such as phytotoxicity, microbial community disruption, and soil health degradation. The framework integrates six core approaches: (i) Strain Selection & Engineering: Prioritize native or stress-adapted strains to ensure environmental compatibility. Multi-strain consortia with complementary functions (e.g., nutrient solubilization + pathogen suppression) enhance resilience. Genetic editing (e.g., CRISPR-Cas9) removes harmful traits (e.g., phytotoxin genes) while preserving beneficial properties. (ii) Application Optimization. Adopt inoculum density to avoid hormonal imbalances (e.g., excessive auxin) and apply during optimal growth stages (e.g., seedling emergence). Use precision delivery methods (e.g., seed coatings) to minimize off-target effects and avoid application during abiotic stress (e.g., drought). (ii) Soil & Environmental Management: Adjust soil pH (6–7) to reduce metal toxicity and stabilize microbial activity. Increase organic matter to buffer nutrient competition and support synergistic interactions. Minimize tillage and synthetic fertilizers to preserve native microbial networks. (iv) Integrated Approaches. Combine PGPB with organic amendments (e.g., biochar) to enhance nutrient retention. Partner with arbuscular mycorrhizal fungi (AMF) to improve root colonization and reduce pathogen pressure. Rotate crops to reset soil microbiomes and disrupt pathogen cycles. (v) Biotechnological Solutions. Engineer strains to degrade phytotoxins (e.g., HCN) or block horizontal gene transfer (e.g., plasmid removal). Use omics-guided formulations (e.g., biofilm encapsulation) to improve survival and targeted root colonization. (vi) Monitoring & Regulation. Pre-screen inoculants for non-target impacts (e.g., biodiversity loss and antibiotic resistance). Implement long-term field surveillance to track soil health and pathogen resistance. Establish policies for strain safety certification and farmer education on context-specific use. These strategies collectively reduce risks while amplifying PGPB benefits, fostering sustainable agroecosystems resilient to climate stressors. By harmonizing microbial innovation with ecological stewardship, this framework ensures plant productivity without compromising soil biodiversity or long-term fertility.

Current inconsistencies in experimental design (e.g., sterilization and duration) limit translatability. Standardized frameworks for reporting soil properties, microbial persistence, and phenotypic outcomes are critical (Azarbad and Junker, 2024). Long-term field trials tracking microbial dynamics across plant developmental stages, coupled with AI-driven predictive models, will optimize consortia design (Correa-Garcia et al., 2023). For instance, phenotyping technologies can non-destructively monitor trait evolution, ensuring inoculant benefits extend to harvest stages (Zieschank and Junker, 2023). By harmonizing strain selection, application precision, and ecological compatibility, PGPB can be sustainably integrated into agriculture, minimizing adverse effects while maximizing crop resilience and productivity (Fig. 4).

## 9 Predicting strain behavior under dynamic environmental conditions

Advances in multi-omics technologies—genomics, transcriptomics, proteomics, and metabolomics—provide transformative insights into the dynamic behavior of PGPB. These tools enable precise predictions of strain-specific traits and their interactions with plants and ecosystems, paving the way for adopting agricultural solutions.

### 9.1 Genomic insights

Whole-genome sequencing (WGS) identifies virulence factors, such as genes encoding toxins (e.g., pyocyanin-encoding genes *phzM* and *phzS* in *Pseudomonas aeruginosa*) (Ortiz-Castro et al., 2014), adhesins, and biofilm-forming proteins (Ferrer-Bustins et al., 2024; Zaghloul and El Halfawy, 2022), ensuring biosafety by excluding pathogenic strains from agricultural use. It also detects antibiotic resistance genes (ARGs), including β-lactamases (*blaTEM*) and efflux pumps (*mexAB-oprM*), critical for assessing horizontal gene transfer risks (Almaghrabi et al., 2024; Holý et al., 2023). Metabolic pathways linked to plant growth promotion are elucidated through WGS, such as *pqq* (phosphate solubilization), *sid* (siderophore biosynthesis), and *acdS* (ACC deaminase for ethylene stress mitigation), alongside stress tolerance genes like *sodB* (superoxide dismutase) and *katA* (catalase) (Gupta et al., 2014). For instance, *Pantoea agglomerans* ANP8, isolated from alfalfa root nodules, harbors *ipdC* (indole-3-acetic acid biosynthesis), *gcd* (glucose dehydrogenase for phosphate solubilization), and salinity tolerance genes (*nhaA* and *nhaK*) (Noori et al., 2021). Similarly, *Bacillus subtilis* BS87 and *B. megaterium* BM89, from sugarcane rhizospheres, possess trehalose metabolism genes (*trehalose-6-phosphate hydrolase*) and glycine betaine biosynthesis operons (*opuA-D*), alongside biocontrol genes like *phzC/F/O* (phenazine synthesis) and *chi113* (chitinase) (Chandra et al., 2021). Heavy metal-resistant *Streptomyces* sp. Z38 encodes a chromate reductase (*MUT89074.1*) for Cr(VI) detoxification and silver nanoparticle biosynthesis genes (*MUT90739.1*, nitrate reductase) (Dávila Costa et al., 2020), while *Streptomyces albireticuli* MDJK11 and *S. alboflavus* MDJK44 feature desferrioxamine B (*desABCD*) for iron chelation, terpene synthases (*2-methylisoborneol*), and polyketide synthases (*candicidin*) for antifungal activity (Wang et al., 2018a). Comparative genomics further identifies core colonization genes, such as *cheA* (chemotaxis) and *luxI/R* (quorum sensing), enhancing root colonization efficacy (Daniels et al., 2004; Scharf et al., 2016; Tang et al., 2020). Despite these advances, challenges persist in distinguishing functional genes from cryptic sequences and validating field performance under fluctuating conditions. Integrating these genomic insights with phenotypic data allows for the rational selection of PGPB strains with optimal beneficial traits while minimizing ecological risks, thereby advancing the development of optimized, sustainable biofertilizers. Such precision in microbial characterization underscores the transformative role of WGS in bridging the gap between genomic potential and functional application in agro-biotechnology.

### 9.2 Transcriptomic and proteomic profiling

Transcriptomic and proteomic profiling are powerful tools for elucidating how PGPB adapt to environmental stressors such as drought, salinity, and temperature fluctuations. These approaches enable the identification of key genes and proteins involved in stress responses, providing insights into molecular mechanisms that underpin survival and functional efficacy (Li et al., 2014a; Wang et al., 2023; Yaghoubi Khanghahi et al., 2022). For example, studies on the Gram-positive bacterium *Bacillus subtilis* demonstrate how these multi-omics approaches can reveal the complex adaptive mechanisms employed by the strain in response to simultaneous stresses, such as nutrient limitations and osmotic challenges. By analyzing gene expression and protein production, researchers can identify key metabolic pathways and regulatory networks that contribute to the strain's resilience. This predictive capability is essential for understanding how PGPB can thrive in fluctuating environments, ultimately informing the development of bioinoculants that enhance plant growth and stress tolerance in agricultural settings (Kohlstedt et al., 2014). Similarly, transcriptomic studies have highlighted global changes in gene expression patterns in microbes during stress exposure, such as those observed in *Streptococcus thermophilus* during pH-controlled fermentations (Qiao et al., 2019). By integrating these multi-omics datasets, researchers can pinpoint biomarkers for stress tolerance and optimize PGPB strains for agricultural applications, ensuring enhanced plant resilience under challenging environmental conditions (Chen et al., 2022). Such advancements are critical for developing sustainable strategies to combat abiotic stresses in crop systems.

### 9.3 Metabolomic predictions

Metabolomic predictions are a powerful tool for identifying both beneficial and toxic metabolites produced by PGPB under specific environmental conditions (Masand et al., 2018). These predictions are crucial for understanding the complex interactions between PGPB and their environments, which can significantly impact plant health and soil ecology. For instance, metabolomic profiling of *Streptomyces* species has demonstrated their ability to synthesize antimicrobial compounds that effectively suppress plant pathogens (Betancur et al., 2020). However, these same compounds may inadvertently inhibit beneficial microbiota, underscoring the dual nature of secondary metabolites in ecological interactions (Bending and Lincoln, 2000). This duality highlights the importance of carefully managing PGPB applications to avoid disrupting beneficial microbial communities. By analyzing metabolic shifts, researchers can predict how PGPB-derived metabolites influence plant resilience and microbial community dynamics (Ferreira et al., 2024). This predictive capability is essential for developing strategies that enhance plant growth and stress tolerance while maintaining a balanced soil microbiome. Integrating metabolomics with metagenomics provides a more comprehensive understanding of rhizosphere metabolite changes induced by PGPB inoculation (Li et al., 2023). This integration aids in the design of synthetic microbial consortia that optimize beneficial effects while minimizing antagonistic interactions. Such consortia can be adapted to specific environmental and crop needs, enhancing the efficacy of PGPB applications. These approaches are critical for advancing sustainable agriculture. By adapting PGPB applications to specific environmental conditions and crop requirements, researchers can promote plant health and productivity while preserving soil biodiversity. This optimized approach not only supports sustainable agricultural practices but also contributes to the development of resilient agroecosystems capable of withstanding environmental stressors. In conclusion, metabolomic predictions, especially when combined with other omics technologies, offer valuable insights into the complex interactions between PGPB, plants, and soil microbiomes. The knowledge is instrumental in designing effective and sustainable agricultural interventions that harness the full potential of PGPB.

## 10 Biosafety and regulatory strategies for PGPB use

Ensuring the safe environmental release of PGPB requires stringent biosafety assessments and regulatory frameworks (Keswani et al., 2019). Current regulations regarding microorganism-bearing products in soil-plant systems are insufficient to adequately address the risks associated with gene transfer and antibiotic resistance (Vassileva et al., 2022). To enhance safety, several key areas require more attention. First, commercial biofertilizers should undergo screening for the presence of ARGs and MGEs prior to approval for agricultural use (Mahdi et al., 2022; Vassileva et al., 2022). Additionally, there is a need for robust biosafety frameworks that establish biosecurity and biocontainment strategies for the use of genetically modified microbes in the environment, particularly those developed through synthetic biology approaches (Ke et al., 2021). Finally, implementing monitoring systems to track the dissemination and persistence of ARGs in agricultural environments where biofertilizers are applied is essential for ensuring long-term safety and efficacy (Checcucci et al., 2020; Vassileva et al., 2022).

Deep screening efforts have identified that some PGPR strains, particularly those belonging to genera like *Serratia, Stenotrophomonas, Burkholderia, Enterobacter, Acinetobacter*, and the *Bacillus cereus* group, possess pathogenic potential and are classified as Biosafety Level 2 (BSL-2) organisms, posing risks as opportunistic human or animal pathogens (Berg et al., 2005; Keswani et al., 2019). Traditional characterization methods, such as small subunit rRNA gene sequencing alone, are insufficient to determine this risk accurately. Therefore, a multilateral polyphasic approach to microbial systematics—integrating phenotypic, chemotaxonomic, and genomic analyses—is essential prior to formulation development and field application to definitively assign risk groups and biosafety levels (Meier-Kolthoff et al., 2013; Prakash et al., 2007). Regulatory strategies must ensure that only risk-group-1 (RG-1) and BSL-1 microorganisms, considered safe for humans and the environment, are approved for commercial biofertilizer formulations and large-scale field use. This includes conducting pathogenicity gene screening and environmental impact assessments to mitigate unintended consequences such as microbial community disruption and the proliferation of antibiotic resistance (Gaiero et al., 2013; Mahdi et al., 2022; Sundh et al., 2011). Conversely, BSL-2 microorganisms should be confined to controlled laboratory research under strict containment conditions that comply with rigorous regulations to prevent unintended environmental release and potential health hazards. In contrast, BSL-1 strains with low-risk profiles should be prioritized for commercial formulations (Selvakumar et al., 2014). Furthermore, comprehensive monitoring of PGPB applications, along with standardized safety indices like the Environmental and Human Safety Index (EHSI), is crucial for ensuring responsible deployment and long-term ecological stability (Nelson, 2004; Vílchez et al., 2016). These strategies highlight the necessity of integrating scientific rigor with regulatory oversight to maximize the agricultural potential of PGPB while maintaining biosafety.

## 11 Future perspectives and research gaps

Future research into PGPB-induced adverse effects must prioritize elucidating molecular mechanisms, such as auxin-ethylene crosstalk and phytotoxic metabolite production (e.g., HCN and VOCs), while establishing toxicity thresholds for compounds like IAA. Long-term ecological studies are critical to assess impacts on soil biodiversity, nutrient cycling, and greenhouse gas emissions across multiple seasons. Advances in strain engineering, including CRISPR-edited PGPB with silenced phytotoxic pathways and optimized microbial consortia, alongside high-throughput screening platforms and nanoencapsulation delivery methods, could enhance efficacy and safety.

A critical frontier lies in understanding the *in situ* evolutionary dynamics of PGPB. Long-term studies tracking genomic and functional shifts in inoculants across diverse soil types and crop rotations are essential to predict key parameters: (1) Adaptive radii – defining the limits of functional divergence strains can undergo while retaining beneficial efficacy; (2) Coevolutionary feedbacks – determining if host plants exert selective pressures that counteract microbial virulence evolution; and (3) Rescue effects – evaluating whether native soil biodiversity can buffer against maladaptive PGPB evolution. Integrating controlled experimental evolution models with field-based meta-omics approaches will elucidate fundamental principles for designing robust, "evolution-resistant" microbial consortia capable of maintaining functional stability under real-world agricultural pressures (Duflos et al., 2024; Mueller and Sachs, 2015).

Understanding context-dependent interactions—such as soil type, climate, and crop genotype—through predictive models will enable tailored applications. Evaluating horizontal gene transfer risks between PGPB and pathogens, alongside biocontainment strategies, is essential to mitigate antibiotic resistance. However, significant gaps persist: limited long-term ecological data, unresolved microbial interaction dynamics, undefined metabolite toxicity thresholds, and inadequate regulatory frameworks for ecotoxicity assessment. To bridge these gaps, interdisciplinary collaboration and multi-omics integration are vital, alongside policy development for standardized risk assessments and farmer-centric decision-support tools. Addressing these challenges will transform PGPB from a paradoxical intervention into a sustainable, predictable solution for global agriculture.

## 12 Conclusion

The dual nature of plant growth-promoting bacteria (PGPB) offers both remarkable potential and significant challenges for sustainable agriculture. While extensively recognized for enhancing nutrient uptake, promoting plant growth, alleviating abiotic stress, and suppressing pathogens, PGPB also pose underappreciated risks. This review highlights a critical but often overlooked paradox: the same microbes recognized for their benefits can, under certain conditions, act as stressors—disrupting plant physiology, altering soil microbial communities, and presenting ecological and biosafety concerns. Although adverse effects of PGPB are less frequently documented—often due to publication bias and context-specific variability—they are real and merit serious consideration. Hormonal imbalances, phytotoxic metabolite production, and antagonistic interactions with native soil microbes exemplify how beneficial strains can become detrimental. Additionally, unintended ecological consequences—such as microbial community disruption, nutrient cycling interference, and the potential for horizontal gene transfer—raise legitimate concerns regarding long-term sustainability and food system safety. The evolutionary plasticity of PGPB, including their capacity to acquire virulence or antibiotic resistance traits, further underscores these risks. These challenges, however, do not diminish the value of PGPB in agriculture. Rather, they underscore the need for a more nuanced, evidence-based strategy for their use. Emerging multi-omics technologies offer promising tools to predict microbial behavior, design safer and more effective microbial consortia, and monitor ecological impacts with high precision. Effective deployment requires careful strain selection, context-specific application rates, integration with broader soil health practices, and adherence to standardized biosafety protocols. Ultimately, the sustainable use of PGPB depends on a deep understanding of their ecological complexity. Bridging current gaps in microbial ecology, host-microbe interactions, and environmental dynamics is essential for balancing agricultural productivity with ecological stewardship. This endeavor demands interdisciplinary collaboration, robust regulatory oversight, and practical tools that support farmer-centered implementation. In summary, PGPB are a cornerstone of sustainable agriculture—but their promise can only be fully realized through a holistic, science-driven, and ecologically informed approach that maximizes benefits while proactively managing risks.

## CRediT authorship contribution statement

**Hassan Etesami:** Writing – review & editing.

## Declaration of competing interest

The authors declare that they have no known competing financial interests or personal relationships that could have appeared to influence the work reported in this paper.

## Data Availability

No data was used for the research described in the article.
